# Genetic analysis reveals candidate genes for activity QTL in the blind Mexican tetra, *Astyanax mexicanus*

**DOI:** 10.7717/peerj.5189

**Published:** 2018-07-18

**Authors:** Brian M. Carlson, Ian B. Klingler, Bradley J. Meyer, Joshua B. Gross

**Affiliations:** 1 Department of Biology, The College of Wooster, Wooster, OH, United States of America; 2 Department of Biological Sciences, University of Cincinnati, Cincinnati, OH, United States of America

**Keywords:** Locomotion, Troglomorphic, Subterranean

## Abstract

Animal models provide useful tools for exploring the genetic basis of morphological, physiological and behavioral phenotypes. Cave-adapted species are particularly powerful models for a broad array of phenotypic changes with evolutionary, developmental and clinical relevance. Here, we explored the genetic underpinnings of previously characterized differences in locomotor activity patterns between the surface-dwelling and Pachón cave-dwelling populations of *Astyanax mexicanus.* We identified multiple novel QTL underlying patterns in overall levels of activity (velocity), as well as spatial tank use (time spent near the top or bottom of the tank). Further, we demonstrated that different regions of the genome mediate distinct patterns in velocity and tank usage. We interrogated eight genomic intervals underlying these activity QTL distributed across six linkage groups. In addition, we employed transcriptomic data and draft genomic resources to generate and evaluate a list of 36 potential candidate genes. Interestingly, our data support the candidacy of a number of genes, but do not suggest that differences in the patterns of behavior observed here are the result of alterations to certain candidate genes described in other species (e.g., teleost multiple tissue opsins, melanopsins or members of the core circadian clockwork). This study expands our knowledge of the genetic architecture underlying activity differences in surface and cavefish. Future studies will help define the role of specific genes in shaping complex behavioral phenotypes in *Astyanax* and other vertebrate taxa.

## Introduction

Since its discovery (Hubbs & Innes, 1936), the blind Mexican tetra, *Astyanax mexicanus*, has proven to be an excellent system for studying the genetic basis of both simple and complex traits (Gross, Meyer & Perkins, 2015). The presence of 29 different cave-adapted populations, that can be interbred with extant surface populations to generate viable hybrid offspring, has rendered *Astyanax* a valuable model in which to investigate evolutionary and developmental phenomena. Included among these features are poorly understood regressive and constructive changes evolving in these and other cave organisms (Jeffery, 2001; Jeffery, 2009). Recently, *Astyanax* and similar species have emerged as natural “evolutionary models” for the study of phenotypes with human medical implications (Albertson et al., 2009), such as craniofacial defects (Gross, Krutzler & Carlson, 2014; Gross et al., 2016b), diseases affecting the eye and retina (O’Quin et al., 2013), biological rhythmicity (Menna-Barreto & Trajano, 2015), sleep (Duboué, Keene & Borowsky, 2011; Duboué & Borowsky, 2012) and behavior (Elipot et al., 2013; Yoshizawa et al., 2015).

“Simpler” organisms have been valuable in exploring the genetics underlying human behavior (Kendler & Greenspan, 2006) and fish species provide an important vertebrate model for understanding neural development, function and disease (Guo, 2004). As a first step toward understanding the genetic basis of traits in any species, it is critical to first identify regions of the genome harboring genes or regulatory sequences that impact phenotypes of interest. This foundation is crucial for narrowing the search to one or more genetic intervals that may be more closely examined. One way to accomplish this is through the use of quantitative trait locus (QTL) analysis (Flint, 2003).

Over the past decade, QTL studies have successfully identified genomic regions associated with a number of behavioral traits. For example, QTL studies have revealed loci associated with boldness in zebrafish (Wright, Butlin & Carlborg, 2006; Wright et al., 2006); feeding, exploration, risk taking, and schooling in species of stickleback (Greenwood et al., 2013; Laine et al., 2014; Greenwood et al., 2015); startle response in medaka (Tsuboko et al., 2014); and anti-predator behavior, response to crowding stress, and spawning/migration behaviors in rainbow trout (Colihueque et al., 2010; Hecht et al., 2012; Rexroad et al., 2013; Christensen et al., 2014). Recent studies carried out in *Astyanax* have identified QTL for feeding angle (shallower feeding angles seen in cavefish are hypothesized to improve foraging success in dark environments; Kowalko et al., 2013a), schooling behavior (loss of schooling behavior in cavefish has been hypothesized to be the result of relaxed selection on schooling in the cave environment; Kowalko et al., 2013b), vibration attraction (increased vibration attraction behavior is a constructive trait that aids cavefish in feeding in the dark; Yoshizawa et al., 2012; Yoshizawa et al., 2015) and locomotor activity (cavefish show a variety of differences in sleep and locomotor activity, relative to surface fish; Yoshizawa et al., 2015).

In this study, we investigated the genetic basis for differences in activity patterns between the surface and cave morphotype of *Astyanax mexicanus*. Analysis of data from 24 hr assays of locomotor activity in an *F*_2_ surface x cavefish hybrid pedigree revealed multiple QTL associated with metrics for overall activity level, as well as spatial components of locomotor activity patterns. We leveraged available genomic and transcriptomic data to screen genes in the genomic intervals underlying these QTL, and generated a set of potential candidates for further study. Our results reveal several genes that may play a heritable role in mediating changes in locomotor activity and related behaviors.

## Materials and Methods

### Fish and animal husbandry

Fish used in these studies were part of a laboratory population maintained at the University of Cincinnati. All specimens were laboratory-bred *Astyanax mexicanus* belonging to lines originally sourced from the Sierra de El Abra region of northeastern Mexico (Gross et al., 2016a; Gross et al., 2016b). *F*_1_ and *F*_2_ hybrids were generated by crossing male surface fish with female (Pachón) cavefish. All fish used in 24 hr activity assays and subsequent QTL analyses were adult fish generously provided to our lab by Dr. Richard Borowsky (New York University).

All fish were kept in a dedicated animal room maintained at 21.7 ±  1 °C under a 12:12hr light/dark cycle (∼160 lux/0 lux). Surface, cave and *F*_1_ hybrid individuals were housed in 18.95 L aquaria on our animal husbandry system as described elsewhere (Gross et al., 2013). *F*_2_ hybrid individuals belonged to a single *F*_2_ mapping pedigree (“Asty66”; *n* = 129) and were reared individually, at room temperature, in 1L tanks filled with water from our husbandry system. Individual housing facilitated identification of individual fish so that phenotypic data could be linked to previously collected genotypic data for each specimen. All fish were fed TetraMin tropical flake food (Tetra) once per day. All husbandry procedures and experimental protocols were approved by the Institutional Animal Care and Use Committee (IACUC) of the University of Cincinnati (Protocol Number 10-01-21-01).

### Automated video tracking

The 24 hr activity assays described in this study were conducted in the same manner and using the same apparatus as described elsewhere (Carlson & Gross, 2018), except as follows: Fish were placed in plastic tanks (30.1 cm wide ×  12.2 cm deep at rim, 28.0 cm wide × 10.7 cm deep at base, 22.5 cm high) filled with approximately 4.5L of water from our husbandry system. These tanks were covered on the back, base and sides by a thin layer of opaque, white mylar to facilitate even distribution of infrared (IR) illumination. One fish was assayed during each 24 hr trial. Trials began at 15 min intervals between zeitgeber time (ZT) 06:00 and 10:00; this allowed time to reset the assay rig between trials, thereby enabling trials to be conducted on successive days.

### Descriptive metrics and statistics

In this study, we chose to characterize locomotor activity based on both velocity (cm/s) and usage of the space within the trial tank (time spent in the top, middle, and bottom thirds of the tank; s/900s time bin for each zone), given that our previous work suggests that patterns in velocity are controlled by an endogenous oscillator in Pachón cavefish, while patterns in spatial tank usage are not (Carlson & Gross, 2018). Three metrics (mean for the entire trial, subjective day mean and subjective night mean) were used to describe velocity and zone usage over the course of a trial as a whole, or specifically during periods when the lights were on or off (ZT 00:00–12:00 and ZT 12:00–00:00, respectively). The former metric provides insights into overall features of a specimen’s activity profile (e.g., high levels of activity or tendency to remain in the bottom zone) and the latter two were chosen to highlight aspects of activity that manifest under certain photic conditions (e.g., decreased velocity in the presence of light or increased use of the top zone when light is absent). Evaluations and metrics relative to circadian rhythmicity (see Carlson & Gross, 2018) were not included in this study as the 24 hr duration of trials did not allow for statistical validation of fit cosine functions or testing of 24 hr time-lag autocorrelation. Instead, a fourth metric, the difference between the mean value for subjective day and subjective night, was used. This metric provides a rough approximation of the relationship between values under light and dark conditions. For example, a low-amplitude diurnal rhythm would result in a small positive value for this metric, while a high-amplitude nocturnal rhythm would result in a larger, negative value.

The presence of albinism in *F*_2_ hybrids was scored as a binary trait based on visual assessment, as was the presence of an eye on the right and left sides of the head. Sex was also scored as a binary trait (1 = female, 0 = male) based on visual assessment of body shape and anal fin morphology (Borowsky, 2008). The sizes of the right and left eye and right and left pupil (in pixels) were measured from images (7.81×  magnification) using ImageJ (National Institutes of Health, Bethesda, MD, USA) according to methods described elsewhere (Gross, Krutzler & Carlson, 2014). Statistical comparisons of velocity and tank usage data between groups based on sex, albinism and presence/absence of eyes were made using Wilcoxon-Mann–Whitney tests in JMP Pro 11 (SAS Institute Inc., Cary, NC, USA).

### Genotyping and quantitative trait locus analyses

Using genomic DNA isolated from tail fin clips (as previously described; Gross et al., 2013), all members of the Asty66 *F*_2_ hybrid pedigree were genotyped during a single round of genotyping-by-sequencing (GBS) conducted at the Cornell University Institute of Biotechnology using previously published methods (Elshire et al., 2011; Lu et al., 2013). Discovery of anonymous SNP markers (contained within 64bp sequences) was completed as part of the GBS procedure. Genotype data for these and other fish were reviewed, suitable markers were identified, and a 2,235 marker, high-density GBS-based linkage map for *Astyanax mexicanus* was constructed (Carlson, Onusko & Gross, 2015). All genetic analyses described in this study are based on this map and genotypic data set.

QTL analyses were conducted in R (R Project for Statistical Computing, http://www.r-project.org/) using the R/qtl package (Broman et al., 2003). Here, we used the scan-one function to perform interval mapping using a single QTL model, as this is the most widely employed method of QTL analysis (Broman & Sen, 2009). Traits with binary or normal distributions were analyzed using each of three scan-one mapping methods: marker regression (MR), expectation maximization (EM), and Haley-Knott (HK), as described elsewhere (Gross, Krutzler & Carlson, 2014). Continuous data with non-normal distributions were subjected to a non-parametric (NP) scan-one analysis. Permutation tests (*n* = 1,000) were used to establish 0.05 *α* LOD significance thresholds for each phenotype-mapping method combination.

### Comparative genomic and transcriptomic analyses

For each of the activity QTL identified, the region of the linkage map associated with the QTL peak was defined as beginning at the marker with the highest LOD value and extending in both directions until either LOD values dropped to a level consistently below the significance threshold, in which case the first marker below the significant LOD value was included, or the end of the linkage group had been reached. In cases where multiple methods returned the same peak marker (or a pseudomarker for which the closest genotyped marker was the same) for a given trait, results from the method that returned the highest LOD score were used to determine this interval. For several QTL, LOD scores dropped below significance for portions of the linkage group, but then “peaked” again further away, suggesting the presence of two or more distinct QTL on a single linkage group. These “secondary peaks” were treated as if they had been separately identified, rather than treating all significant LOD scores within a given region as part of a single peak, which would require inclusion of long stretches of markers without significant association to the phenotype of interest.

Earlier BLAST analyses anchored 598 unplaced, annotated genome scaffolds (McGaugh et al., 2014) to the GBS-based *Astyanax* linkage map by identifying putative locations for 2091 of the 2235 GBS markers that comprised the map (Carlson, Onusko & Gross, 2015). This information facilitated identification of portions of the genome associated with linkage map intervals containing activity QTL. The list of genes on these scaffolds was then used as a starting point for candidate gene identification.

BioMart (v. 0.7) was used to query the Ensembl genome browser (release 78; http://www.ensembl.org) and retrieve gene ontology (GO) terms associated with each of the genes found on *Astyanax* genome scaffolds anchored to the region of our linkage map associated with each QTL peak. In cases where ZFIN IDs for zebrafish homologs were provided, GO annotations for that species were queried as well. Genes annotated using one or more relevant GO terms were considered provisional candidates and subjected to further analysis.

Gene expression was evaluated using RNA-seq data (Stahl & Gross, 2017). This data set included a developmental series consisting of pooled samples from both cave and surface fish at 10 hpf, 24 hpf, 1.5 dpf and 3 dpf (50 embryos per sample, three technical replicates), as well as pooled samples (three biological replicates, two technical replicates) from juvenile (three to four month old) cave and surface fish raised under both 12:12hr light/dark conditions and in total darkness (see description of dark-reared fish in Carlson & Gross, 2018). Total RNA was isolated using an RNeasy Kit (Qiagen; Valencia, CA, USA) and samples were sequenced by the Cincinnati Children’s Hospital Medical Center DNA Sequencing and Genotyping Core facility using Illumina HiSeq technology (v. 2 kit). SeqMan NGen (v. 11; DNAstar, Madison, WI, USA) was used to align RNAseq reads to an *Astyanax* transcriptome template and ArrayStar (v. 11; DNAstar, Madison, WI) was used to normalize read counts using the RPKM method (Mortazavi et al., 2008), calculate fold change comparisons between samples and determine statistical significance using the Student *t*-test controlled for false-discovery rate (Benjamini & Hochberg, 1995). Differences in gene expression between surface and cavefish for genes with relevant GO annotations were then examined.

RNA-seq reads from cave and surface fish were also aligned against the *Astyanax* reference genome (McGaugh et al., 2014) using SeqMan NGen (v. 11; DNAstar, Madison, WI, USA). For each QTL, SNP reports were generated for all scaffolds anchored to the associated interval. Results were then filtered to limit potential variants to those with alleles segregating between cave (Pachón) and surface in all (or nearly all; >90%) reads. Variant calls in genes with relevant GO annotations were examined and manually verified.

**Figure 1:**
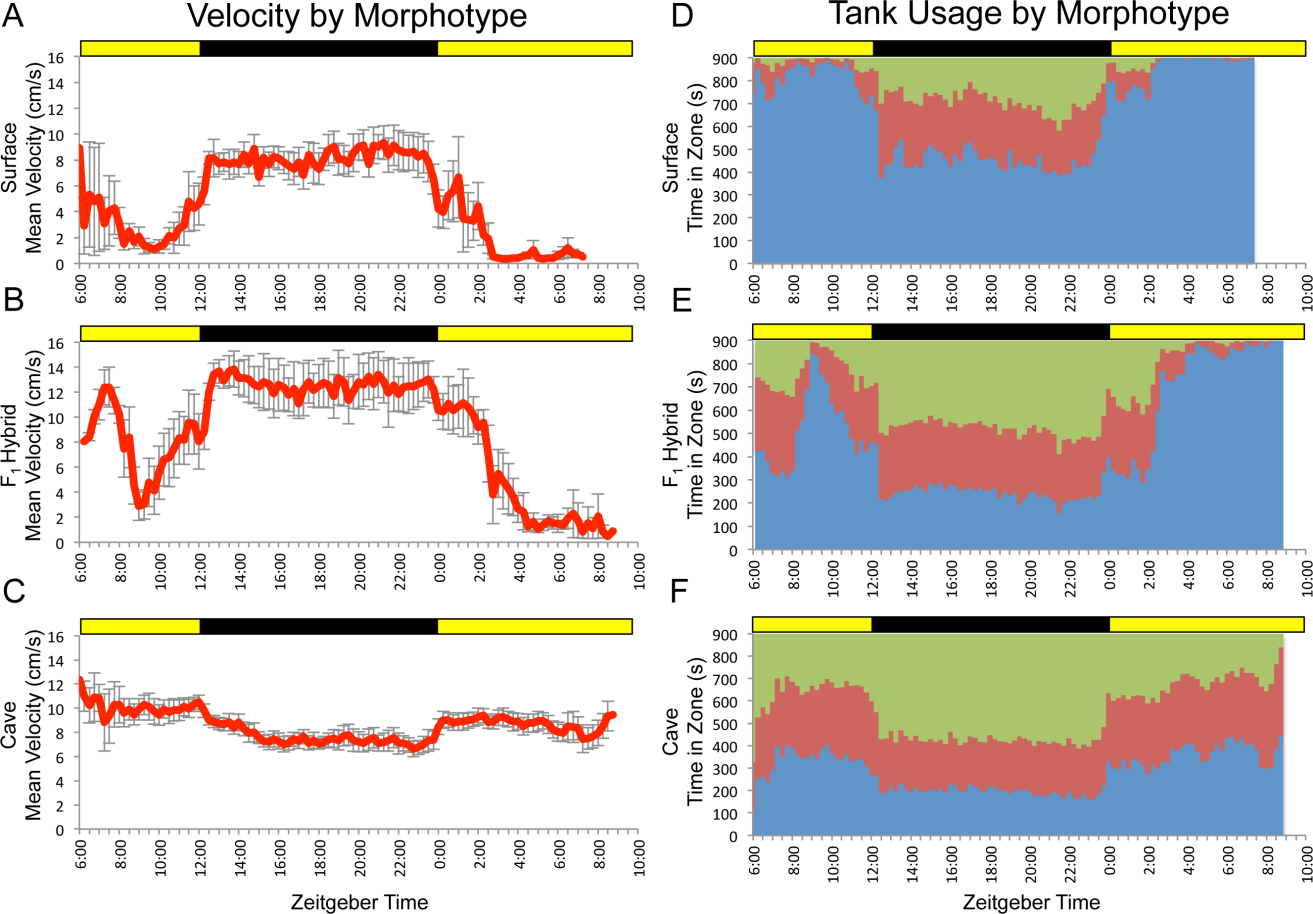
*F*_1_ surface x Pachón hybrids show surface-like activity patterns with cave-like influences. Data shown are mean values for a small number of surface (*n* = 5; A,D), cave (*n* = 8; C,F) and F1 hybrid (*n* = 5; B,E) individuals assayed for 24 hr under 12:12 hr light/dark conditions. Trial starts were staggered, so data shown cover more than 24 hrs and displays greater variability at the very beginning and near the end. Data binned at 15 min (900 s) intervals. Green = time in top zone, red = time in middle zone, blue = time in bottom zone. Times shown are in zeitgeber time (ZT); ZT 00:00 = lights on, ZT 12:00 = lights off. Yellow bars = subjective day, black bars = subjective night.

## Results

### Behavioral analysis

Previously, we demonstrated that the surface and cave (Pachón) morphotypes of *Astyanax mexicanus* exhibit differences in patterns of overall activity, as well as the spatial component of locomotor behavior (Carlson & Gross, 2018). Given that these differences were observed at the population level, we hypothesized that observed activity profiles had a heritable genetic basis. To test this, we assayed a small number of surface, cave and *F*_1_ hybrid fish for 24 hr under 12:12hr light/dark conditions. Our results for surface and cavefish varied slightly from those previously reported (Carlson & Gross, 2018). We attribute these differences to the fact that the fish used in this study were generally older, larger and had substantially more room to swim within the trial tank than those used in previous studies. Generally speaking, *F*_1_ individuals displayed overall patterns similar to surface fish, but with “cave-like” influences, such as higher mean velocity and a less extreme bias in usage of the bottom zone (Fig. 1). This mirrors the fact that, morphologically, *F*_1_ hybrids look like surface fish, but with subtle differences that make them slightly more cave-like (e.g., smaller eyes and reduced pigmentation; Sadoglu, 1956).

**Figure 2:**
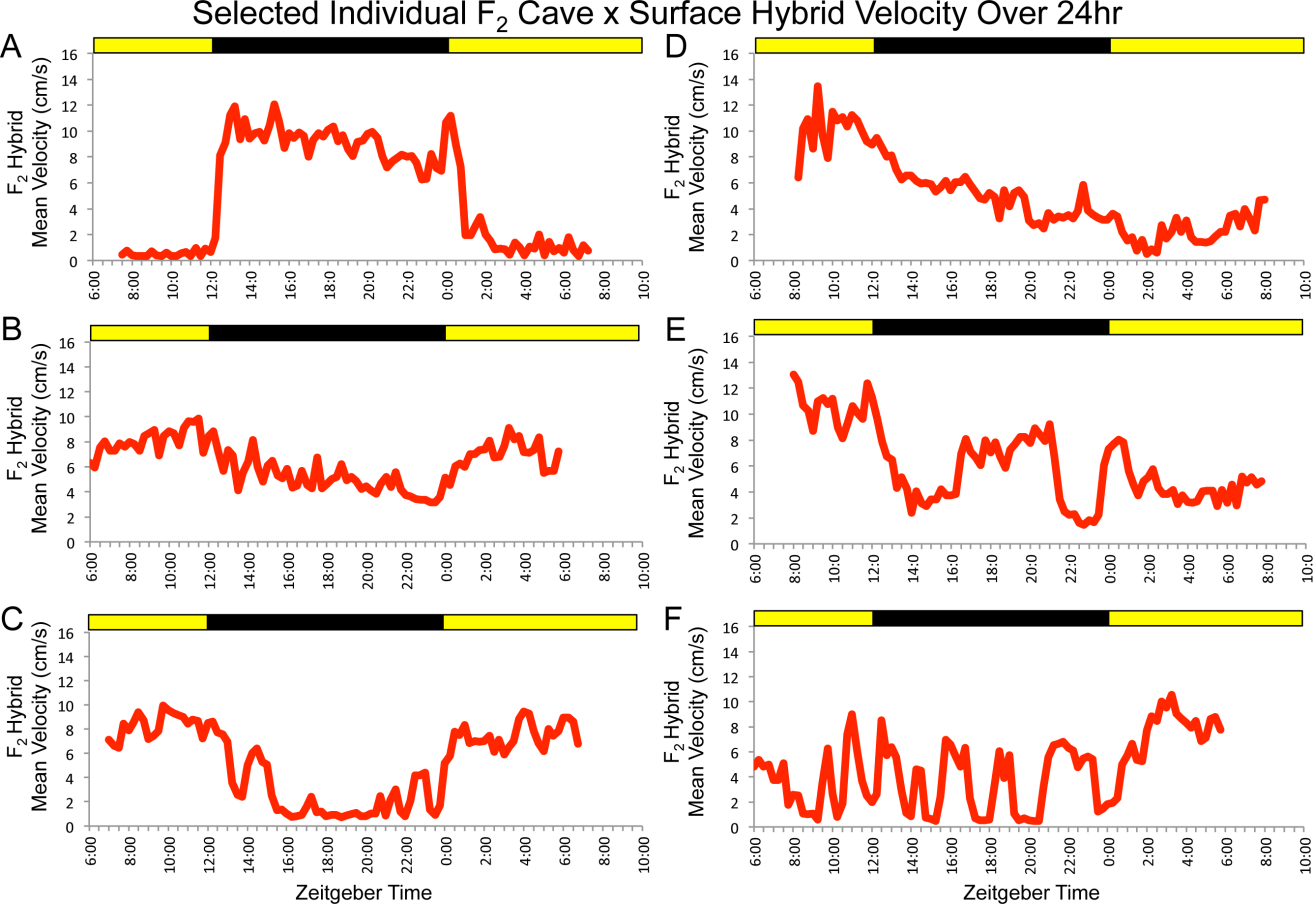
*F*_2_ surface x Pachón hybrids show a wide array of parental and non-parental activity patterns. Some individuals show activity profiles that are similar to surface or cavefish (A and B, respectively) or that combine elements of both (C). Others display patterns of activity that do not resemble those of either parental morphotype (e.g., D–F). Each panel includes data for one F2 individual assayed for 24 hr under 12:12 hr light/dark conditions. Trial starts were staggered. Times shown are in zeitgeber time (ZT); ZT 00:00 = lights on, ZT 12:00 = lights off. Yellow bars = subjective day, black bars = subjective night.

We then assayed a large *F*_2_ hybrid population under the same conditions. *F*_2_ surface x cavefish hybrid pedigrees typically include individuals that appear similar to surface fish (i.e., pigmented individuals with two eyes), some that look like cavefish (i.e., albino individuals lacking eyes), and several individuals with intermediate phenotypes resulting from various combinations of cave- and surface-like traits (e.g., albino fish with two eyes, pigmented fish with no eyes, and fish that show various levels of reduction, asymmetry or disorganization in eyes and pigmentation; Sadoglu, 1956). In the same way, we observed a wide range of locomotor activity patterns in our *F*_2_ pedigree. Some fish displayed activity profiles similar to one of the parental populations, others showed a clear combination of elements of the behavior of both surface and cavefish and many showed patterns that were not recognizable as belonging to either morphotype (Fig. 2). These results are indicative of the complex nature of locomotor activity and suggest that the patterns of activity (or lack thereof) displayed by individual specimens are influenced by a number of different genetic loci.

### QTL analysis

**Figure 3:**
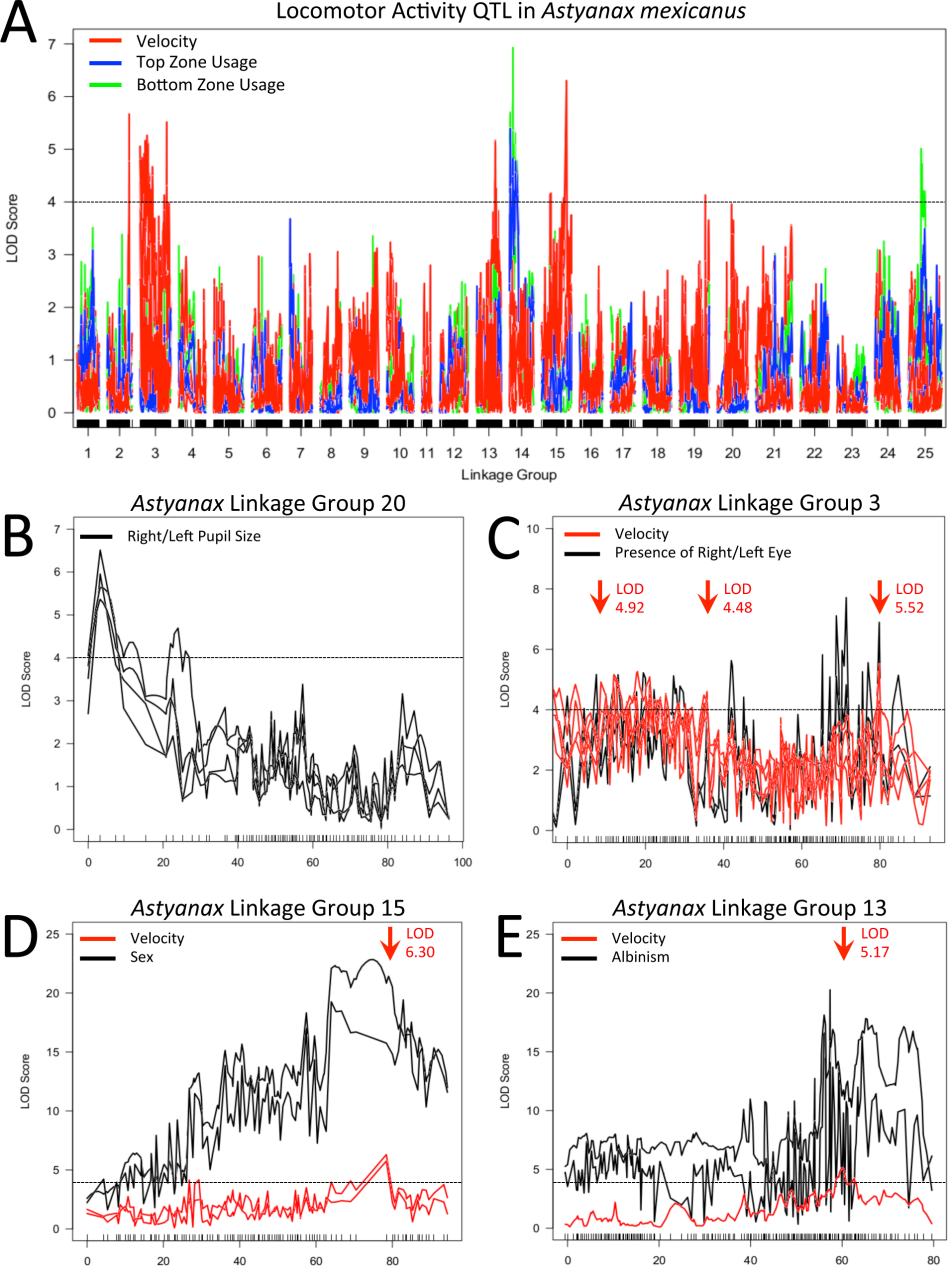
Locomotor activity QTL examined. Velocity (red), top usage (blue) and bottom usage (green) QTL data are shown with the results of multiple mapping methods included where the location of QTL peaks differed between methods (A). QTL for right/left pupil size (LG 20; B), presence/absence of right/left eye (LG 3; C), sex (LG15; D) and presence/absence of albinism (LG 13; E) are shown in subsequent panels, with co-localizing activity QTL included. In all panels, arrows indicate the location of primary or secondary (a single peak at 35 cM on LG 3) QTL intervals examined after evaluating effect plots for each peak seen in our data. Dotted black lines represent a LOD threshold of 4.0, above which peaks are considered potentially relevant, but not necessarily statistically significant. All indicated peaks have significant LOD scores based on permutation tests calculated for the particular combination of activity metric and mapping method that are represented.

In order to determine if a genetic basis for our activity measures could be identified, we conducted QTL analyses using data from members of our *F*_2_ surface x cave (Pachón) hybrid pedigree, each assayed for 24 h under a 12:12 light/dark cycle (Table S1 ; genotypic data provided in Carlson, Onusko & Gross, 2015). Our analysis revealed a number of putative associations between regions of our linkage map and metrics for both mean velocity and tank usage (Table 1). At least one QTL was found for each of three different velocity metrics and five different tank usage metrics, with QTL distributed over six linkage groups from our GBS-based linkage map (Carlson, Onusko & Gross, 2015). In several instances, our efforts to determine the interval covered by a given QTL revealed that there were two or more distinct LOD “peaks” on a given linkage group. In these cases, secondary peaks were investigated independently, as if identified on different linkage groups. We investigated overlap between our behavioral QTL and regions of the genome associated with presence/absence of eyes, eye size and pupil size on either side of the head, as well as albinism and sex (Fig. 3; Table S2). As suggested by previous studies (Duboué, Keene & Borowsky, 2011; Yoshizawa et al., 2015), we found no correspondence between our behavioral results and QTL for the size of the eye or pupil on either the right or left side; we found no significant associations with eye size and QTL for pupil size were confined to linkage group 20. However, when scored as a binary trait, QTL for presence/absence of an eye on either side of the head were found on linkage group 3 near a QTL for mean velocity during subjective day. Statistical analysis shows that there is a significant difference between the mean “day” velocity of *F*_2_ hybrids that have a right (*z* = 2.677, *p* = 0.0074) or left (*z* = 3.227, *p* = 0.0013) eye and those who do not. Similarly, the QTL for albinism previously described on linkage group 13 of this map (Carlson, Onusko & Gross, 2015) has its peak LOD score at a marker very close to another QTL peak for mean “day” velocity; scores for this activity metric differ significantly between albino and non-albino members of our *F*_2_ pedigree (*z* = 3.417, *p* = 0.0006).

**Table 1:** Results of QTL analysis for metrics of locomotor activity. Results based on 24 hr assay data for *Astyanax* surface x Pachón F_2_ hybrids (*n* = 127) under 12:12 hr light/dark conditions. For all listed peaks, *p* ≤ 0.05 based on 1,000 permutations.

Data set	Method^a^	Activity metric^b^	Peak marker	Linkage group^c^	Position (cM)	LOD score	Closest marker	Variance explained
Velocity	MR	Day Mean	TP73890	2	69.50	5.67	–	18.6%
Velocity	EM	Day Mean	TP73890	2	69.50	4.76	–	15.9%
Velocity	HK	Day Mean	TP73890	2	69.50	4.63	–	15.5%
Velocity	MR	Trial Mean	TP74449	3	−4.05	5.06	–	16.8%
Velocity	HK	Trial Mean	TP74449	3	−4.05	4.82	–	16.0%
Velocity	EM	Trial Mean	TP60697	3	8.60	4.92	–	16.3%
Velocity	EM	Day Mean	TP86516	3	17.90	5.25	–	17.3%
Velocity	HK	Day Mean	TP86516	3	17.90	5.27	–	17.4%
Velocity	MR	Day Mean	TP11322	3	79.80	5.52	–	18.1%
Velocity	EM	Day Mean	TP44190	13	60.00	5.17	–	17.1%
Velocity	HK	Day Mean	TP44190	13	60.00	5.14	–	17.0%
Top Usage	NP	Day Mean	TP65410	14	2.60	5.39	–	17.8%
Bottom Usage	NP	Day Mean	TP65410	14	2.60	5.70	–	18.7%
Bottom Usage	EM	Trial Mean	TP53209	14	10.60	6.09	–	19.8%
Bottom Usage	HK	Trial Mean	TP53209	14	10.60	5.95	–	19.4%
Bottom Usage	MR	Trial Mean	TP19556	14	10.90	6.93	–	22.2%
Top Usage	NP	Trial Mean	TP36991	14	19.40	4.55	–	15.2%
Velocity	MR	Night Mean	TP73771	15	78.40	6.30	–	20.4%
Velocity	EM	Night Mean	TP73771	15	78.40	5.19	–	17.2%
Velocity	HK	Night Mean	TP73771	15	78.40	5.42	–	17.8%
Velocity	MR	Trial Mean	TP73771	15	78.42	5.76	–	18.8%
Velocity	HK	Trial Mean	TP73771	15	78.42	4.64	–	15.5%
Velocity	EM	Trial Mean	c15.loc79	15	79.00	4.51	TP73771	15.1%
Bottom Usage	HK	Day Minus Night	c25.loc40	25	40.00	5.01	TP57986	16.6%
Bottom Usage	EM	Day Minus Night	c25.loc43	25	43.00	4.99	TP9488	16.6%
Bottom Usage	MR	Day Minus Night	TP9488	25	43.10	4.96	–	16.5%

**Notes.**

a MR, Marker Regression; EM, Expectation Maximization; HK, Haley-Knott; NP, Non-Parametric

b “Day” refers to the period when lights were on (ZT 00:000-12:00); “night” refers to the period when lights were off (ZT 12:00-00:00).

c Linkage groups listed are from the map presented in Carlson, Onusko & Gross (2015).

In addition to potential links with cave-associated traits, the LOD peak of a robust QTL for sex on linkage group 15 is situated one marker away from the peak values of QTL for both mean velocity throughout entirety of the trial and mean velocity during subjective night. Statistical analyses indicate a significant difference between male and female members of our *F*_2_ pedigree for both mean trial velocity (*z* =  − 2.751, *p* = 0.0059) and mean “night” velocity (*z* =  − 3.362, *p* = 0.0008).

**Table 2:** Results of gene ontology-based candidate gene screen. Listed GO terms based on annotations of *Astyanax* genes and/or zebrafish homologs.

Ensembl gene ID	Gene symbol	Gene name	*Astyanax* scaffold	GO term
ENSAMXG00000000759	*col15a1b*	*collagen, type XV, alpha 1b*	KB871754.1	Eye development
ENSAMXG00000000885	*xcr1a.1 (1 of 2)*	*chemokine (C motif) receptor 1a, duplicate 1*	KB871754.1	Locomotion
ENSAMXG00000000886	*xcr1a.1 (2 of 2)*	*chemokine (C motif) receptor 1a, duplicate 1*	KB871754.1	Locomotion
ENSAMXG00000000888	*cc2d2a*	*coiled-coil and C2 domain containing 2A*	KB871754.1	Photoreceptor cell outer segment organization
ENSAMXG00000005193	*cxcl32b.1*	*chemokine (C-X-C motif) ligand 32b, duplicate 1*	KB871766.1	Locomotion
ENSAMXG00000001564	*znf503 (1 of 2)*	*zinc finger protein 503*	KB872296.1	Embryonic camera-type eye morphogenesis
ENSAMXG00000002007	*pitx3*	*paired-like homeodomain 3*	KB872296.1	Camera-type eye development
ENSAMXG00000002007	*pitx3*	*paired-like homeodomain 3*	KB872296.1	Lens development in camera-type eye
ENSAMXG00000002296	*sfrp5*	*secreted frizzled-related protein 5*	KB872296.1	Eye development
ENSAMXG00000012711	*dmbx1b*	*diencephalon/mesencephalon homeobox 1b*	KB882082.1	Retina development in camera-type eye
ENSAMXG00000013049	*vapb*	*VAMP (vesicle-associated membrane protein)-associated protein B and C*	KB882082.1	Swimming behavior
ENSAMXG00000013417	novel gene	Uncharacterized protein	KB882082.1	Locomotion
ENSAMXG00000014705	*prickle1a*	*prickle homolog 1a (Drosophila)*	KB882082.1	Neural retina development
ENSAMXG00000014945	*cerkl*	*ceramide kinase-like*	KB882082.1	Photoreceptor cell development
ENSAMXG00000014945	*cerkl*	*ceramide kinase-like*	KB882082.1	Retina layer formation
ENSAMXG00000009797	*mcm3*	*minichromosome maintenance complex component 3*	KB882090.1	Retina development in camera-type eye
ENSAMXG00000010179	*opn5*	*opsin 5*	KB882090.1	Photoreceptor activity
ENSAMXG00000021415	*igf1rb*	*insulin-like growth factor 1b receptor*	KB882097.1	Camera-type eye development
ENSAMXG00000021415	*igf1rb*	*insulin-like growth factor 1b receptor*	KB882097.1	Embryonic camera-type eye development
ENSAMXG00000021574	*lim2.2*	*lens intrinsic membrane protein 2.2*	KB882097.1	Structural constituent of eye lens
ENSAMXG00000021593	*uhrf1*	*ubiquitin-like with PHD and ring finger domains 1*	KB882097.1	Lens development in camera-type eye
ENSAMXG00000019838	*crb2a*	*crumbs family member 2a*	KB882104.1	Camera-type eye development
ENSAMXG00000019838	*crb2a*	*crumbs family member 2a*	KB882104.1	Embryonic retina morphogenesis in camera-type eye
ENSAMXG00000020264	*htr2cl2*	*5-hydroxytryptamine (serotonin) receptor 2C, G protein-coupled-like 2*	KB882104.1	Feeding behavior
ENSAMXG00000020264	*htr2cl2*	*5-hydroxytryptamine (serotonin) receptor 2C, G protein-coupled-like 2*	KB882104.1	Locomotory behavior
ENSAMXG00000005992	novel gene	uncharacterized protein	KB882105.1	Regulation of behavior
ENSAMXG00000010609	*lepr*	*leptin receptor*	KB882115.1	Camera-type eye development
ENSAMXG00000012018	*fgf8*	*fibroblast growth factor 8*	KB882125.1	Embryonic camera-type eye development
ENSAMXG00000012018	*fgf8*	*fibroblast growth factor 8*	KB882125.1	Embryonic retina morphogenesis in camera-type eye
ENSAMXG00000012018	*fgf8*	*fibroblast growth factor 8*	KB882125.1	Retina development in camera-type eye
ENSAMXG00000012130	*chata*	*choline O-acetyltransferase a*	KB882125.1	Locomotory behavior
ENSAMXG00000012172	*rgra*	*retinal G protein coupled receptor a*	KB882125.1	Phototransduction
ENSAMXG00000012172	*rgra*	*retinal G protein coupled receptor a*	KB882125.1	Locomotion
ENSAMXG00000012545	novel gene	uncharacterized protein	KB882125.1	Locomotion
ENSAMXG00000002537	*b9d2*	*B9 protein domain 2*	KB882129.1	Opsin transport
ENSAMXG00000005363	*rb1*	*retinoblastoma 1*	KB882129.1	Optic nerve development
ENSAMXG00000005363	*rb1*	*retinoblastoma 1*	KB882129.1	Retinal ganglion cell axon guidance
ENSAMXG00000005527	*dlat*	*dihydrolipoamide S-acetyltransferase*	KB882129.1	Detection of light stimulus
ENSAMXG00000005527	*dlat*	*dihydrolipoamide S-acetyltransferase*	KB882129.1	Detection of light stimulus involved in visual perception
ENSAMXG00000025967	*atoh7*	*atonal homolog 7*	KB882154.1	Camera-type eye development
ENSAMXG00000025967	*atoh7*	*atonal homolog 7*	KB882154.1	Eye development
ENSAMXG00000025967	*atoh7*	*atonal homolog 7*	KB882154.1	Retina layer formation
ENSAMXG00000025967	*atoh7*	*atonal homolog 7*	KB882154.1	Swimming behavior
ENSAMXG00000008135	*tmtopsb*	*teleost multiple tissue opsin b*	KB882155.1	Photoreceptor activity
ENSAMXG00000008135	*tmtopsb*	*teleost multiple tissue opsin b*	KB882155.1	Phototransduction
ENSAMXG00000016819	*scinla*	*scinderin like a*	KB882172.1	Eye development
ENSAMXG00000002437	novel gene	uncharacterized protein	KB882228.1	Photoreceptor activity
ENSAMXG00000011539	*rgs4*	*regulator of G-protein signaling 4*	KB882253.1	Locomotory behavior
ENSAMXG00000014314	*marcksa*	*myristoylated alanine-rich protein kinase C substrate a*	KB882283.1	Retina layer formation
ENSAMXG00000005947	*tfap2a*	*transcription factor AP-2 alpha*	KB882287.1	Retina development in camera-type eye

**Table 3:** Results of gene expression analysis. Listed fold changes are for Pachón cavefish, relative to surface fish. Only genes with significant expression differences for at least one time point are shown.

Ensembl transcript ID	Gene	*Astyanax* scaffold	Fold change at 10 hpf	Fold change at 24 hpf	Fold change at 1.5 dpf	Fold change at 3 dpf	Fold change in L/D juveniles	Fold change in D/D juveniles
ENSAMXT00000000806	*col15a1b*	KB871754.1	1.632 up	2.403 up^*^	1.244 up	1.631 down^*^	1.584 down^*^	1.023 up
ENSAMXT00000000907	*cc2d2a*	KB871754.1	1.269 down	1.419 down^*^	1.599 down	1.217 down^*^	1.206 down	1.195 down
ENSAMXT00000005317	*cxcl32b.1*	KB871766.1	none	15.495 down	37.715 up^*^	44.778 up^*^	1.353 down	1.422 up
ENSAMXT00000001586	*znf503 (1 of 2)*	KB872296.1	1.375 up^*^	1.200 up	1.123 down	1.618 down^*^	1.524 up	1.085 down
ENSAMXT00000002039	*pitx3*	KB872296.1	1.236 up	1.434 down^*^	1.419 down	2.394 down^*^	1.290 up	1.395 down
ENSAMXT00000002347	*sfrp5*	KB872296.1	1.569 up^*^	1.235 up	1.233 up^*^	1.088 up	1.005 down	1.018 up
ENSAMXT00000013064	*dmbx1b*	KB882082.1	1.384 down^*^	1.367 down^*^	1.101 down	2.169 down^*^	1.036 down	3.593 down
ENSAMXT00000013423	*vapb*	KB882082.1	1.306 up^*^	1.010 down	1.113 down	1.223 up^*^	1.114 up	1.007 down
ENSAMXT00000010067	*mcm3*	KB882090.1	1.561 down^*^	1.168 down^*^	1.252 down	1.170 down	1.024 up	1.836 up
ENSAMXT00000010451	*opn5*	KB882090.1	3.679 down	6.341 down^*^	1.933 down	3.681 down^*^	1.170 up	2.169 down
ENSAMXT00000022053	*igf1rb*	KB882097.1	1.537 up	1.278 up	1.299 up	1.528 up^*^	1.101 down	1.242 down
ENSAMXT00000022220	*lim2.2*	KB882097.1	1.228 down	12.428 down	18.190 up	4.197 up	78.344 down	181.240 down^*^
ENSAMXT00000022239	*uhrf1*	KB882097.1	1.029 up	1.230 down^*^	1.202 down	1.079 up	1.582 down	1.475 up
ENSAMXT00000020429	*crb2a*	KB882104.1	1.344 up	1.695 down^*^	1.367 down	1.710 down^*^	4.724 down	7.837 down^*^
ENSAMXT00000020869	*htr2cl2*	KB882104.1	39.977 up	1.096 up	2.188 up	7.862 up^*^	7.454 up	3.285 down
ENSAMXT00000010901	*lepr*	KB882115.1	1.493 up	1.463 down	1.060 up	1.209 down^*^	1.008 down	1.157 down
ENSAMXT00000012355	*fgf8*	KB882125.1	1.311 down	1.635 up^*^	1.360 up	1.419 up^*^	1.339 up	1.018 down
ENSAMXT00000012506	*chata*	KB882125.1	35.993 up^*^	1.350 up	1.204 up	1.298 down	1.268 down	2.173 down
ENSAMXT00000012519	*rgra*	KB882125.1	2.550 up^*^	2.003 up^*^	1.077 up	2.655 down^*^	1.552 up	1.043 down
ENSAMXT00000012896	novel gene	KB882125.1	3.288 up^*^	6.177 up^*^	5.560 up	4.456 up^*^	1.339 up	2.353 up
ENSAMXT00000002596	*b9d2*	KB882129.1	1.265 down^*^	1.140 down	1.129 down	1.439 down	1.755 down	1.908 down
ENSAMXT00000005728	*dlat*	KB882129.1	1.696 up	1.061 down	1.197 down	1.209 up^*^	1.141 up	1.283 up
ENSAMXT00000026693	*atoh7*	KB882154.1	none	4.119 up	38.702 down	11.083 down^*^	74.041 down^*^	1.044 down
ENSAMXT00000017315	*scinla*	KB882172.1	1.387 down^*^	2.026 up^*^	1.167 up	1.123 down	1.178 down	1.280 down
ENSAMXT00000011876	*rgs4*	KB882253.1	2.272 up^*^	1.204 up^*^	1.919 up^*^	2.004 up^*^	1.385 down	1.806 down
ENSAMXT00000014726	*marcksa*	KB882283.1	1.427 down^*^	1.096 down	1.208 down	1.248 down^*^	1.019 up	1.006 up
ENSAMXT00000006092	*tfap2a*	KB882287.1	1.692 down^*^	1.259 down^*^	1.169 down	1.253 down^*^	1.722 up	1.431 down

**Notes.**

TITLE Hpf hours post fertilization dpf days post fertilization

* *p* ≤ 0.05.

**Table 4:** Results of sequence variation analysis.

Ensembl ID	Gene	*Astyanax* scaffold	Portion of gene	Genomic change	Protein change
ENSAMXG00000000759	*col15a1b*	KB871754.1	CDS	g.69818A>G	–
ENSAMXG00000000759	*col15a1b*	KB871754.1	Intron	g.81811G>C	–
ENSAMXG00000000885	*xcr1a.1 (1 of 2)*	KB871754.1	CDS	g.1085A>C	p.His277Pro
ENSAMXG00000000888	*cc2d2a*	KB871754.1	CDS	g.1407T>C	–
ENSAMXG00000000888	*cc2d2a*	KB871754.1	CDS	g.1883A>G	–
ENSAMXG00000000888	*cc2d2a*	KB871754.1	CDS	g.3139G>A	–
ENSAMXG00000000888	*cc2d2a*	KB871754.1	CDS	g.13847G>A	–
ENSAMXG00000000888	*cc2d2a*	KB871754.1	CDS	g.20242G>A	–
ENSAMXG00000000888	*cc2d2a*	KB871754.1	CDS	g.20272G>A	–
ENSAMXG00000001564	*znf503 (1 of 2)*	KB872296.1	3′UTR	g.89A>G	–
ENSAMXG00000001564	*znf503 (1 of 2)*	KB872296.1	3′UTR	g.399A>G	–
ENSAMXG00000001564	*znf503 (1 of 2)*	KB872296.1	3′UTR	g.487T>A	–
ENSAMXG00000001564	*znf503 (1 of 2)*	KB872296.1	3′UTR	g.492C>T	–
ENSAMXG00000001564	*znf503 (1 of 2)*	KB872296.1	CDS	g.2184G>A	p.Ala262Val
ENSAMXG00000001564	*znf503 (1 of 2)*	KB872296.1	5′UTR	g.3498T>C	–
ENSAMXG00000001564	*znf503 (1 of 2)*	KB872296.1	5′UTR	g.3570A>G	–
ENSAMXG00000002007	*pitx3*	KB872296.1	CDS	g.1056G>A	–
ENSAMXG00000002296	*sfrp5*	KB872296.1	3′UTR	g.25305A>T	–
ENSAMXG00000012711	*dmbx1b*	KB882082.1	CDS	g.4566C>T	–
ENSAMXG00000009797	*mcm3*	KB882090.1	3′UTR	g.8193T>G	–
ENSAMXG00000021415	*igf1rb*	KB882097.1	CDS	g.88C>T	–
ENSAMXG00000021415	*igf1rb*	KB882097.1	CDS	g.16248C>T	p.Arg793Gln
ENSAMXG00000021415	*igf1rb*	KB882097.1	CDS	g.21599G>A	–
ENSAMXG00000021415	*igf1rb*	KB882097.1	CDS	g.24682A>C	–
ENSAMXG00000019838	*crb2a*	KB882104.1	5′UTR	g.2318G>T	–
ENSAMXG00000019838	*crb2a*	KB882104.1	CDS	g.5815C>T	–
ENSAMXG00000019838	*crb2a*	KB882104.1	CDS	g.5894A>G	p.Val1014Ala
ENSAMXG00000019838	*crb2a*	KB882104.1	CDS	g.9101A>G	–
ENSAMXG00000019838	*crb2a*	KB882104.1	CDS	g.10605C>T	p.Glu557Lys
ENSAMXG00000019838	*crb2a*	KB882104.1	CDS	g.12316G>A	–
ENSAMXG00000019838	*crb2a*	KB882104.1	CDS	g.12484G>T	–
ENSAMXG00000019838	*crb2a*	KB882104.1	CDS	g.17234C>T	–
ENSAMXG00000019838	*crb2a*	KB882104.1	CDS	g.18107A>C	p.Ser58Ala
ENSAMXG00000010609	*lepr*	KB882115.1	5′UTR	g.7A>G	–
ENSAMXG00000010609	*lepr*	KB882115.1	CDS	g.10195G>T	–
ENSAMXG00000010609	*lepr*	KB882115.1	3′UTR	g.24538T>G	–
ENSAMXG00000012172	*rgra*	KB882125.1	3′UTR	g.2534G>A	–
ENSAMXG00000002537	*b9d2*	KB882129.1	CDS	g.3893G>A	–
ENSAMXG00000005363	*rb1*	KB882129.1	CDS	g.2798C>T	–
ENSAMXG00000005363	*rb1*	KB882129.1	Intron	g.15718C>A	–
ENSAMXG00000005527	*dlat*	KB882129.1	CDS	g.11697C>A	–
ENSAMXG00000025967	*atoh7*	KB882154.1	CDS	g.95T>A	p.Tyr110Phe
ENSAMXG00000011539	*rgs4*	KB882253.1	Intron	g.2193G>A	–
ENSAMXG00000014314	*marcksa*	KB882283.1	Intron	g.1615C>G	–
ENSAMXG00000005947	*tfap2a*	KB882287.1	Intron	g.5998G>C	–

**Notes.**

CDS Coding sequence UTR Untranslated region

### Identification of candidate genes

Having identified a number of genomic regions putatively associated with various elements of the activity profiles observed in our 24 hr assays, we sought to explore candidate genes located in these intervals. We focused on genes underlying QTL peaks with phenotypic effect plots that showed a clear genetic effect; those QTL that were not investigated typically had effect plots that revealed a high degree of variability among specimens possessing two “surface” alleles. As a result, we examined a total of eight intervals in our map, all but one of which contained a primary QTL peak for at least one locomotor activity trait. These intervals ranged from 1.587 to 10.001 cM in length (mean = 4.286 cM) and contained between three and seven GBS markers (mean = 4.5 markers). The sequences for the markers in each interval were used to anchor between two and six genomic scaffolds (mean = 3.375 scaffolds), thereby associating 3.256–20.761 Mb (mean = 9.086 Mb) of genomic sequence and 75–413 genes (mean = 192.75 genes) with each interval examined. We therefore began our analysis with an initial list of 1542 genes distributed over 72.685 Mb of the *Astyanax* genome.

To reduce this list to a more manageable size, we screened for genes with relevant gene ontology (GO) terms. We were particularly interested in genes associated with GO terms relating to locomotion, swimming, detection of (or response to) light stimulus, and circadian rhythms. Additionally, given the likelihood that ability to perceive and respond to lighting cues is affected by the presence/absence of eyes, we also included genes annotated with GO terms related to the development and maintenance of the eye and associated structures. This initial screen condensed the list of potential candidates to 36 genes (Table 2). These genes were then examined for both sequence variation and differences in expression between morphs. RNA-seq data revealed statistically significant differential expression in 27 of these genes, with the majority of expression differences occurring only during development (Table 3). Only three genes showed significant differential expression in both juvenile samples and the developmental series and a single gene showed differential expression only in juveniles, however this disparity is likely influenced by a difference in the number of technical replicates between developmental and juvenile samples. Analysis of sequence variation based on alignment of RNA-seq reads to the *Astyanax* draft genome (McGaugh et al., 2014) identified 16 genes that include exonic SNPs with alleles segregating between surface and Pachón cave samples. Thirteen genes possessed one or more variants in the coding sequence and six genes showed variation in the 5′- or 3′-untranslated regions (UTR; Table 4). There were no obvious indels or splice variations observed in any of these genes. Of the 13 genes showing variation in the coding sequence, only five genes possessed non-synonymous changes. Interestingly, five genes showed variation in regions annotated as introns, three of which are genes unique to this category. This may be due to either incorrect alignment or retained introns in uncharacterized splice variants. Taken together, these analyses provide some level of additional support for 29 of the 36 genes identified by screening GO terms. A summary of these results is provided in Table 5.

**Table 5:** Summary of candidate gene assessment. For specific details, refer to Table 2–Table 4.

Ensembl ID	Gene	*Astyanax* scaffold	Relevant GO terms?	Differential Expression		Sequence Variation		
				Development	Juvenile	5′ or 3′ UTR	Synonymous	Non- synonymous
ENSAMXG00000000759	*col15a1b*	KB871754.1	Yes	Yes	Yes	No	Yes	No
ENSAMXG00000000885	*xcr1a.1 (1 of 2)*	KB871754.1	Yes	No	No	No	No	Yes
ENSAMXG00000000886	*xcr1a.1 (2 of 2)*	KB871754.1	Yes	No	No	No	No	No
ENSAMXG00000000888	*cc2d2a*	KB871754.1	Yes	Yes	No	No	Yes	No
ENSAMXG00000005193	*cxcl32b.1*	KB871766.1	Yes	Yes	No	No	No	No
ENSAMXG00000001564	*znf503 (1 of 2)*	KB872296.1	Yes	Yes	No	Yes	No	Yes
ENSAMXG00000002007	*pitx3*	KB872296.1	Yes	Yes	No	No	Yes	No
ENSAMXG00000002296	*sfrp5*	KB872296.1	Yes	Yes	No	Yes	No	No
ENSAMXG00000012711	*dmbx1b*	KB882082.1	Yes	Yes	No	No	Yes	No
ENSAMXG00000013049	*vapb*	KB882082.1	Yes	Yes	No	No	No	No
ENSAMXG00000013417	novel gene	KB882082.1	Yes	No	No	No	No	No
ENSAMXG00000014705	*prickle1a*	KB882082.1	Yes	No	No	No	No	No
ENSAMXG00000014945	*cerkl*	KB882082.1	Yes	No	No	No	No	No
ENSAMXG00000009797	*mcm3*	KB882090.1	Yes	Yes	No	Yes	No	No
ENSAMXG00000010179	*opn5*	KB882090.1	Yes	Yes	No	No	No	No
ENSAMXG00000021415	*igf1rb*	KB882097.1	Yes	Yes	No	No	Yes	Yes
ENSAMXG00000021574	*lim2.2*	KB882097.1	Yes	No	Yes	No	No	No
ENSAMXG00000021593	*uhrf1*	KB882097.1	Yes	Yes	No	No	No	No
ENSAMXG00000019838	*crb2a*	KB882104.1	Yes	Yes	Yes	Yes	Yes	Yes
ENSAMXG00000020264	*htr2cl2*	KB882104.1	Yes	Yes	No	No	No	No
ENSAMXG00000005992	novel gene	KB882105.1	Yes	No	No	No	No	No
ENSAMXG00000010609	*lepr*	KB882115.1	Yes	Yes	No	Yes	Yes	No
ENSAMXG00000012018	*fgf8*	KB882125.1	Yes	Yes	No	No	No	No
ENSAMXG00000012130	*chata*	KB882125.1	Yes	Yes	No	No	No	No
ENSAMXG00000012172	*rgra*	KB882125.1	Yes	Yes	No	Yes	No	No
ENSAMXG00000012545	novel gene	KB882125.1	Yes	Yes	No	No	No	No
ENSAMXG00000002537	*b9d2*	KB882129.1	Yes	Yes	No	No	Yes	No
ENSAMXG00000005363	*rb1*	KB882129.1	Yes	No	No	No	Yes	No
ENSAMXG00000005527	*dlat*	KB882129.1	Yes	Yes	No	No	Yes	No
ENSAMXG00000025967	*atoh7*	KB882154.1	Yes	Yes	Yes	No	No	Yes
ENSAMXG00000008135	*tmtopsb*	KB882155.1	Yes	No	No	No	No	No
ENSAMXG00000016819	*scinla*	KB882172.1	Yes	Yes	No	No	No	No
ENSAMXG00000002437	*opn7a*	KB882228.1	Yes	No	No	No	No	No
ENSAMXG00000011539	*rgs4*	KB882253.1	Yes	Yes	No	No	No	No
ENSAMXG00000014314	*marcksa*	KB882283.1	Yes	Yes	No	No	No	No
ENSAMXG00000005947	*tfap2a*	KB882287.1	Yes	Yes	No	No	No	No

## Discussion

### Genetic analyses reveal a complex genetic basis for activity differences between cave and surface fish

The identification of multiple genetic loci associated with aspects of locomotor activity in this species indicates the complexity of the genetic underpinnings of these behaviors and of the alterations seen in cavefish, relative to surface populations. Interestingly, our results also suggest that while mean velocity and tank usage are under genetic control, different regions of the genome mediate these aspects of locomotor behavior. Linkage groups 2, 3, 13 and 15 harbor QTL for at least one velocity metric; linkage groups 14 and 25 harbor QTL for at least one tank usage metric. This demonstrates the value of evaluating activity level (e.g., mean velocity) and the spatial component of locomotor behavior (e.g., time spent in different zones) independently, rather than dealing with combined metrics (Erckens & Weber, 1976; Erckens & Martin, 1982a; Erckens & Martin, 1982b) or employing methods that do not account for spatial components of activity (Thines & Wolff-Van Ermengem, 1965; Thines & Weyers, 1978; Duboué, Keene & Borowsky, 2011; Beale et al., 2013; Yoshizawa et al., 2015).

### Co-localized QTL may indicate relationships between patterns in locomotor behavior, sex and cave-associated traits

It has long been theorized that there is a link between regression of the visual system and loss of rhythmic behavior in cave species. In a recent review of over 40 cave-adapted species, Friedrich (2013) noted that behaviorally arrhythmic species are almost universally “primary anophthalmic” species (i.e., species that never develop an eye); adult-specific and population-specific anophthalmic species appear to retain some level of behavioral rhythmicity. Acknowledging the potential role that eye loss and other cave-associated traits may play in influencing patterns in locomotor activity, recent studies have looked for associations between activity data and traits such as eye size, pupil size, thickness of the inner nuclear layer of the retina, and albinism (Duboué, Keene & Borowsky, 2011; Yoshizawa et al., 2015). Additionally, studies examining other behavioral phenotypes in this species have explored potential links between sex and the behaviors observed (Yoshizawa, Ashida & Jeffery, 2012; Elipot et al., 2013; Kowalko et al., 2013b). With the exception that tendency to school was shown to differ between sexes in a surface x cave (Tinaja) *F*_2_ hybrid population (Kowalko et al., 2013b), no other such links have been shown between these factors and behavior in this species.

While the exact nature of the relationship between eye loss, albinism and the behavioral QTL described in this study remains unclear, any association beyond simple linkage due to proximity is likely influenced by selective pressures acting on either the morphological trait, the associated behavioral trait, or both. Initially, it was theorized that there may be selective pressure to lose morphological features rendered useless in the darkness, perhaps as an advantage conferred by energy conservation (Poulson & White, 1969; Culver, 1982). However, the current body of literature does not seem to support this hypothesis in the case of the loss/reduction of either eyes (Jeffery, 2005) or pigmentation (Bilandžija et al., 2013). Instead, it appears more likely that the loss of eyes and pigmentation is associated with beneficial morphological, physiological and/or behavioral changes and that positive selective pressures acting on the latter are responsible for indirect selection upon the former (see Wright, 1964). For example, a link between eye reduction and increases in vibration attraction behavior has been proposed (Yoshizawa et al., 2012), although the precise nature of this connection is a matter of some debate (Borowsky, 2013). Additionally, Bilandžija et al. (2013) suggest that albinism may be the result of selection upon behavioral changes that result from down-regulation of *oca2* and the associated increase in tyrosine and catecholamine levels that occurs in the absence of melanin synthesis. Therefore, potential links between changes in locomotor activity patterns such as those presented in this study and regressive cave-associated traits provide excellent opportunities to further examine the mechanisms underlying the constellation of constructive and regressive traits evolving in these and other cave-adapted species.

Our results also suggested a link between patterns of locomotor activity and sex. While this connection is noteworthy and should be kept in mind during experimental design, it is more likely that it is the result of sex-based differences in behavioral traits such as boldness (Dahlbom et al., 2011; Irving & Brown, 2013; King et al., 2013; Ingley, Rehm & Johnson, 2014), rather than a consequence of some aspect of cave adaptation.

### QTL for locomotor activity are distinct from those previously identified

A recent study by Yoshizawa et al. (2015) presented two distinct QTL associated with locomotor activity. The results presented in that study are based on the linkage map published by O’Quin et al. (2013), which does not share any markers with the linkage map used in this study. Direct comparison of QTL results between the current study and that of Yoshizawa et al. (2015), while desirable, is therefore impossible. However, it is possible to make inferences about the relationships between QTL presented in these two studies by using the draft *Astyanax* genome (McGaugh et al., 2014) as a reference, as described elsewhere (Carlson, Onusko & Gross, 2015). When the locations of genomic scaffolds that can be anchored to both maps are compared, clear relationships between the linkage groups comprising each of these two linkage maps emerge (Table S3). Consequently, it appears likely that the QTL for locomotor activity reported on linkage groups 3 and 22 by Yoshizawa et al. (2015) correspond with regions of linkage groups 7/8 and 5, respectively, in the map employed here. Given that none of the QTL identified in this study are found on those linkage groups, it is reasonable to assume that the QTL described in these two studies represent the influence of different loci on patterns in locomotor activity.

### Comparative genomic and transcriptomic analyses identify putative candidate genes mediating differential activity in cavefish

The approach employed here has certain limitations, namely that it cannot identify genes on genomic scaffolds that were not anchored to the examined intervals of our linkage map, or genes that are not (yet) associated with relevant GO terms. Further, it does not enable us to examine potential regulatory mutations in sequences outside of transcribed regions, or differences in gene expression beyond the specific time points we profiled. However, while these limitations mean that our list of potential candidates is far from exhaustive, we believe that our analysis does provide evidence that further examination into the potential role of several genes in mediating locomotor differences is warranted. These particularly strong candidates are discussed below:

*Regulator of G-protein signaling 4* (*rgs4*), found on a scaffold anchored to the region underlying the QTL on linkage group 13, was the only gene to be expressed at significantly different levels across all four time points in our developmental series. Our analysis suggests that *rgs4* is likely overexpressed in cavefish, relative to surface fish, throughout development. In zebrafish, *rgs4* has been shown to play a role in axonogenesis of neurons involved in motor activity and responses to touch (Cheng et al., 2013). In other species, it has also been shown to play a role in serotonin signaling (Gu, Jiang & Yan, 2007) and modulation of melatonin receptor signaling in retinal ganglion cells (Ji et al., 2011). Given the importance of melatonin in sleep and circadian rhythmicity (Iuvone et al., 2005; Gandhi et al., 2015), the proposed role of serotonin signaling in the cavefish “behavioral syndrome” (Elipot et al., 2013; Elipot et al., 2014) and the differences in locomotor activity seen here, further investigation into the potential effects of elevated *rgs4* in cavefish is warranted.

**Figure 4:**
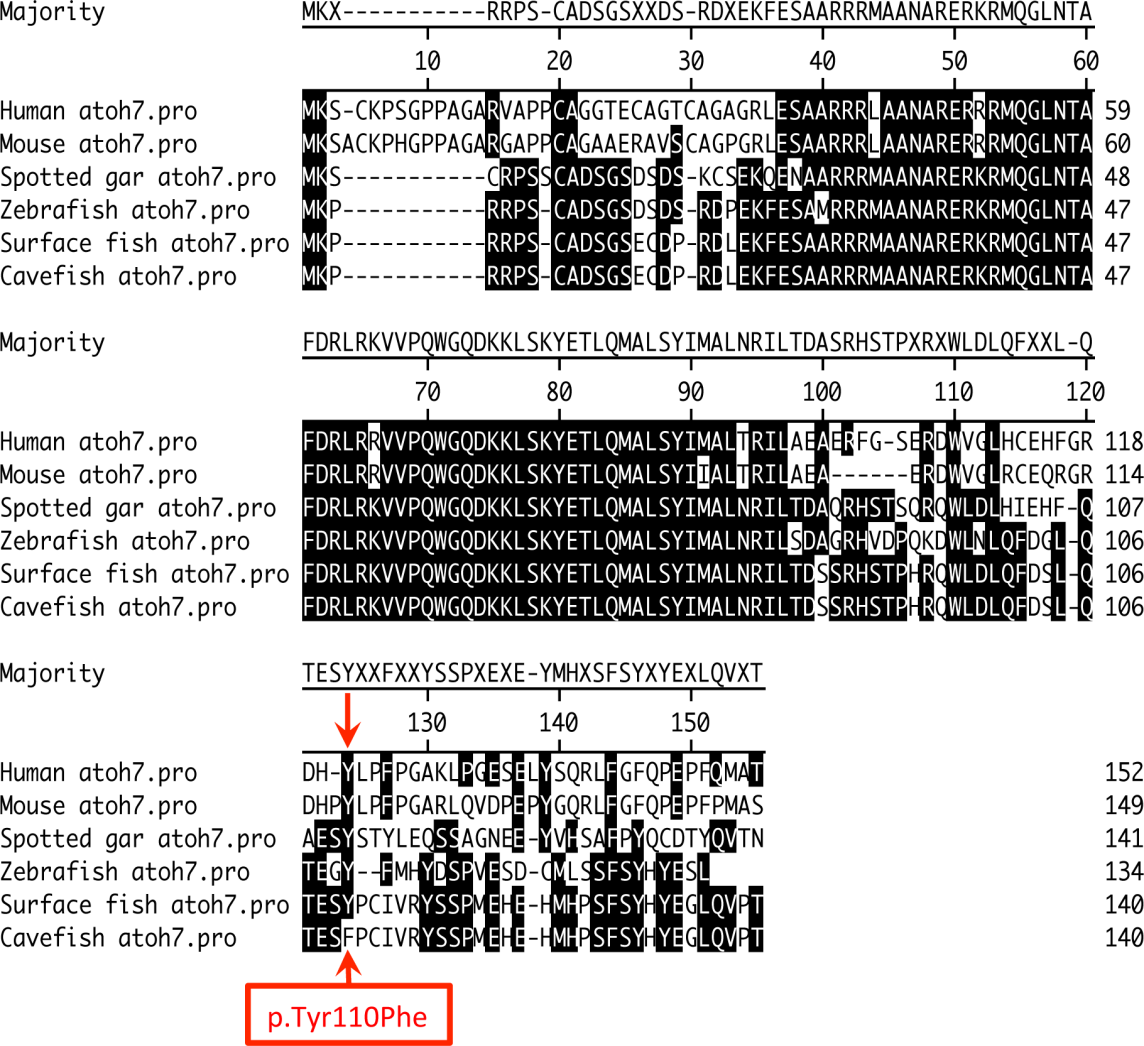
Non-synonymous mutation causes amino acid substitution at highly conserved position in cavefish ATOH7. Amino acids that match the consensus are shaded in black. The potential effect of this mutation has not been functionally evaluated.

Similarly, *atonal homolog 7* (*atoh7*), which is found on a scaffold anchored to the interval underlying the QTL on linkage group 25, stands out as a potential candidate due to the fact that, out of the five genes in which non-synonymous sequence variation was observed, *atoh7* contained the only mutation in which cavefish display an amino acid change at a highly conserved amino acid residue (Fig. 4). Additionally, this gene is significantly under-expressed in cavefish at 3 dpf, as well in juveniles reared under 12:12hr light/dark conditions. No difference in expression was observed between surface and cavefish juveniles reared under constant darkness. In zebrafish, *atoh7* (formerly known as *lakritz* or *atonal homolog 5*) has been shown to be essential for differentiation of retinal ganglion cells (Kay et al., 2001). Functionally blind *atoh7* null mutant zebrafish larvae also show small but significant defects in certain locomotor behaviors (Mirat et al., 2013). Further analysis could elucidate the effects, if any, of the *atoh7* sequence variation and expression differences observed in Pachón cavefish.

*Opsin 5* (*opn5*; *neuropsin*) is found on a scaffold anchored to the critical region underlying a QTL for mean day velocity on linkage group 3 and is significantly under-expressed in cavefish at 24 hpf and 3 dpf. The protein product of the *opn5* gene is UV-sensitive in zebrafish (Yamashita et al., 2014), although there is little information about its temporal expression or function in zebrafish. Manchenkov et al. (2015) noted “developmental phenotypic defects” in zebrafish subjected to morpholino knockdown of *opn5*, but they did not elaborate upon the nature of these defects. In other species, *opn5* is present in the retina and skin, has been implicated in deep brain photoreception, and is found in both the pineal gland and serotonin-positive cells in the hypothalamus (Yamashita et al., 2014; Haltaufderhyde et al., 2015). The presence of *opn5* in the retina, pineal gland and serotonin-positive cells in the hypothalamus, together with the general lack of information about its expression patterns and function in fish, renders this gene a prime candidate for further examination.

While the above candidates were particularly well supported, the following results were also noteworthy:

(1) *Insulin-like growth factor 1b receptor* (*igf1rb*), *crumbs family member 2a* (*crb2a*) and *zinc finger protein 503* (*znf503*) each contain multiple SNPs with alleles segregating between surface and Pachón cave samples, including at least one non-synonymous mutation, and show significant differential expression during at least one developmental stage. They may therefore warrant further investigation.

(2) *Fibroblast growth factor 8* (*fgf8*) is present on a scaffold anchored within the QTL interval on linkage group 25 and shows significant overexpression in cavefish, relative to surface fish at 24 hpf and 3 dpf. Elevated levels of this gene have been implicated in retinal defects in *Astyanax* (Pottin, Hinaux & Rétaux, 2011).

(3) *Choline O-acetyltransferase a* (*chata*), located on the same genomic scaffold as *fgf8*, is highly over-expressed in cavefish at 10hpf. In zebrafish, *chata* plays a part in the role of acetylcholine as an important neuromodulator in early development; cells in the brain and retina as well as the optic nerve and spinal motorneurons are ChAT-immunoreactive from various early stages of development (Arenzana et al., 2005). Additionally, the zebrafish *bajan* mutant line shows compromised motility and fatigue as a result of a point mutation in the *chata* gene (Wang, Wen & Brehm, 2008). Therefore, it is possible that the early over-expression of *chata* seen in the Pachón population may contribute to the increased level of activity observed in cavefish.

(4) *Teleost multiple tissue opsin b* (*tmtopsb*) is present in our data set but shows no evidence of sequence variation or significant expression differences. Additionally, none of the four known cavefish *melanopsin* (*opn4*) genes appear to be anchored within the QTL intervals examined here. Truncation of members of these two opsin families has been demonstrated in *Phreatichthys andruzzii* and implicated in that species’ inability to entrain locomotor activity with exogenous lighting cues (Cavallari et al., 2011). Beale et al. (2013) also found no evidence to implicate these genes in the behavioral alterations observed in *Astyanax*.

(5) Similarly, none of the genes present on scaffolds anchored to the QTL intervals examined here are part of the core circadian clockwork or the stabilizing loop described in zebrafish (Vatine et al., 2011), nor were any of the genes annotated with GO terms indicating an obvious role in maintenance of circadian rhythms.

(6) Recent work by Jaggard et al. (2018) implicates hypocretin/orexin (HCRT) in the evolution of sleep loss in Pachón cavefish. While sleep was not specifically assayed in this study and *hypocretin (orexin) neuropeptide precursor* (*hcrt*) was not included in the gene set analyzed here, it is worth noting that this gene is found on a genomic scaffold that has been anchored to several positions along linkage group 2 (KB882213.1; Carlson, Onusko & Gross, 2015). One of these anchor points is a marker that is nearby, though not within, the QTL interval for mean day velocity found on that linkage group in this study.

## Conclusions

In this study, we identified several novel QTL underlying patterns of locomotor activity in *Astyanax mexicanus.* Further, our results suggest that spatial components of locomotor activity, such as patterns in usage of the top and bottom of the trial tank, have a genetic basis that is distinct from loci underlying patterns in the overall level of activity captured by velocity measurements. Finally, examination of the relevant genetic intervals using a combination of genomic and transcriptomic data enabled us to build a list of potential candidate genes for further study in this species and highlight several genes with particularly strong support. The results presented here serve to further our understanding of the genetic underpinnings of locomotor activity patterns in this species and lay the groundwork for additional studies elucidating how particular genes contribute to the development, maintenance or modulation of complex behaviors in vertebrate taxa.

##  Supplemental Information

10.7717/peerj.5189/supp-1 Table S1 Activity data for Asty66 *F*_2_ surface x Pachón hybrid pedigree assayed for 24 hr under 12:12 hr light/dark conditions Here *n* = 127; two specimens were deceased before assays could be conducted.

10.7717/peerj.5189/supp-2 Table S2 Sex, albinism and eye data for Asty66 *F*_2_ surface x Pachón hybrid pedigree All members of the pedigree (*n* = 129) were scored for sex (1 = female, 0 = male), presence of albinism and presence of right and left eye (1 = present, 0 = absent) in a binary fashion. Eye and pupil size measurements (in pixels) were made from images where possible; in some cases an eye was technically present, but was too disorganized to measure the eye and/or pupil accurately.

10.7717/peerj.5189/supp-3 Table S3 Distribution of commonly-anchored genomic scaffolds between linkage maps published by Carlson, Onusko & Gross (2015) and O’Quin et al. (2013) Numbers indicate the number of genomic scaffolds anchored to both indicated linkage groups.

## Additional Information and Declarations

### Data Availability

The following information was supplied regarding data availability: All sequencing reads are available through the NCBI Sequence Reads Archive under BioProject accession PRJNA258661.
