# Supplementary material for: Genetic analysis reveals candidate genes for activity QTL in the blind Mexican tetra, Astyanax mexicanus
Source: PeerJ. 2018 Jul 18;6:e5189. doi: 10.7717/peerj.5189 (PMC6054784; doi:10.7717/peerj.5189)
Supplement: Table S1 — Here n = 127; two specimens were deceased before assays could be conducted. [file peerj-06-5189-s001.docx]

**Table S1: Activity data for Asty66 F_2_ surface x Pachón hybrid pedigree assayed for 24hr under 12:12hr light/dark conditions.** Here n = 127; two specimens were deceased before assays could be conducted.

| **Specimen** | **Velocity Data (cm/s)** | | | | **Top Zone Usage Data (s/900s)** | | | | **Bottom Zone Usage Data (s/900s)** | | | |
| --- | --- | --- | --- | --- | --- | --- | --- | --- | --- | --- | --- | --- |
|  | **Trial Mean** | **Day Mean** | **Night Mean** | **Day - Night** | **Trial Mean** | **Day Mean** | **Night Mean** | **Day - Night** | **Trial Mean** | **Day Mean** | **Night Mean** | **Day - Night** |
| Asty66-001 | 7.295445491 | 7.939838938 | 6.651052044 | 1.288786893 | 233.9377566 | 243.1215929 | 224.7539203 | 18.36767263 | 289.8589554 | 289.4943545 | 290.2235563 | -0.729201833 |
| Asty66-002 | 3.701063739 | 0.603390786 | 6.798736693 | -6.195345907 | 253.0172531 | 2.152150813 | 503.8823554 | -501.7302046 | 495.4739467 | 882.5659004 | 108.3819931 | 774.1839073 |
| Asty66-003 | 7.479609775 | 4.963537137 | 9.995682413 | -5.032145276 | 129.1823078 | 17.61761785 | 240.7469977 | -223.1293799 | 573.7817669 | 750.9711097 | 396.5924242 | 354.3786854 |
| Asty66-004 | 7.987692302 | 6.079070386 | 9.896314219 | -3.817243833 | 193.3099765 | 25.64161356 | 360.9783395 | -335.3367259 | 452.6213725 | 757.8349183 | 147.4078266 | 610.4270917 |
| Asty66-005 | 5.648295587 | 1.819395915 | 9.477195259 | -7.657799344 | 67.50083419 | 0.119564 | 134.8821044 | -134.7625404 | 656.5951375 | 893.1952785 | 419.9949965 | 473.200282 |
| Asty66-006 | 2.291887594 | 2.541555256 | 2.042219933 | 0.499335322 | 255.5972645 | 56.70323233 | 454.4912966 | -397.7880643 | 461.09547 | 713.7588957 | 208.4320444 | 505.3268513 |
| Asty66-007 | 4.91287673 | 6.089907894 | 3.735845567 | 2.354062327 | 202.5685402 | 154.9931871 | 250.1438933 | -95.15070619 | 540.6740076 | 574.1964189 | 507.1515964 | 67.0448225 |
| Asty66-008 | 6.441225124 | 3.468676118 | 9.413774129 | -5.945098011 | 76.16018845 | 16.17867881 | 136.1416981 | -119.9630193 | 686.6449792 | 827.0965425 | 546.1934159 | 280.9031266 |
| Asty66-009 | 4.069436972 | 3.69973399 | 4.439139955 | -0.739405965 | 142.8160791 | 133.9130791 | 151.7190791 | -17.80600006 | 405.641058 | 466.2544485 | 345.0276676 | 121.226781 |
| Asty66-010 | 8.225417118 | 7.237583625 | 9.213250612 | -1.975666988 | 108.314912 | 57.87801652 | 158.7518075 | -100.873791 | 542.2352904 | 625.3329726 | 459.1376082 | 166.1953644 |
| Asty66-011 | - | - | - | - | - | - | - | - | - | - | - | - |
| Asty66-012 | 3.062399852 | 1.879301217 | 4.245498487 | -2.36619727 | 156.5211043 | 16.89328229 | 296.1489263 | -279.255644 | 551.4504086 | 681.7866478 | 421.1141695 | 260.6724784 |
| Asty66-013 | 2.214045068 | 2.671073912 | 1.757016224 | 0.914057689 | 158.4160547 | 127.4740014 | 189.358108 | -61.88410663 | 499.4508402 | 616.3726239 | 382.5290565 | 233.8435673 |
| Asty66-014 | 6.314358674 | 5.686666858 | 6.94205049 | -1.255383632 | 186.4993478 | 125.009734 | 247.9889615 | -122.9792275 | 382.8714137 | 449.3966182 | 316.3462093 | 133.0504089 |
| Asty66-015 | 4.913681553 | 5.091289525 | 4.736073581 | 0.355215944 | 223.4832053 | 192.038567 | 254.9278436 | -62.8892766 | 492.7528233 | 487.1364438 | 498.3692029 | -11.23275917 |
| Asty66-016 | 6.544809552 | 6.742451276 | 6.347167829 | 0.395283447 | 128.9098122 | 187.4047654 | 70.41485908 | 116.9899063 | 549.9155405 | 496.591034 | 603.240047 | -106.649013 |
| Asty66-017 | 4.836573959 | 0.937097322 | 8.736050596 | -7.798953274 | 23.09809829 | 1.399315958 | 44.79688063 | -43.39756467 | 743.9317794 | 851.9874043 | 635.8761545 | 216.1112498 |
| Asty66-018 | 7.618853397 | 8.759593276 | 6.478113518 | 2.281479758 | 144.3752781 | 110.5786336 | 178.1719225 | -67.59328885 | 537.0669282 | 564.2017025 | 509.9321539 | 54.26954858 |
| Asty66-019 | 4.120807061 | 1.793147898 | 6.448466223 | -4.655318325 | 272.9396052 | 4.945917875 | 540.9332925 | -535.9873746 | 476.471611 | 842.2255592 | 110.7176629 | 731.5078963 |
| Asty66-020 | 6.037611369 | 4.577961233 | 7.497261505 | -2.919300271 | 207.9555251 | 169.8636135 | 246.0474368 | -76.18382333 | 354.7043565 | 365.7317023 | 343.6770107 | 22.05469165 |
| Asty66-021 | 4.544219412 | 1.595263448 | 7.493175376 | -5.897911928 | 65.0143198 | 0.619369375 | 129.4092702 | -128.7899009 | 753.6241102 | 894.5445444 | 612.7036761 | 281.8408683 |
| Asty66-022 | 2.5452212 | 1.251795578 | 3.838646821 | -2.586851242 | 31.94826785 | 6.526665417 | 57.36987029 | -50.84320488 | 812.5066038 | 866.497748 | 758.5154597 | 107.9822882 |
| Asty66-023 | 3.216902417 | 2.775351303 | 3.658453531 | -0.883102228 | 361.3599706 | 144.6522913 | 578.0676499 | -433.4153585 | 259.9884609 | 414.6396402 | 105.3372817 | 309.3023585 |
| Asty66-024 | 4.883081335 | 1.903605219 | 7.862557452 | -5.958952233 | 123.1321596 | 11.8264095 | 234.4379097 | -222.6115002 | 634.973168 | 839.4672461 | 430.4790899 | 408.9881562 |
| Asty66-025 | 4.913911574 | 1.373173706 | 8.454649441 | -7.081475735 | 170.1204673 | 9.754893542 | 330.486041 | -320.7311475 | 549.7400178 | 861.8569971 | 237.6230386 | 624.2339585 |
| Asty66-026 | 11.88369531 | 10.37734355 | 13.39004707 | -3.012703522 | 165.6837388 | 42.79209608 | 288.5753816 | -245.7832855 | 525.1397231 | 680.9629086 | 369.3165375 | 311.6463711 |
| Asty66-027 | 6.295022977 | 7.00919289 | 5.580853064 | 1.428339826 | 147.8419389 | 90.84223092 | 204.8416468 | -113.9994159 | 460.569945 | 547.6469521 | 373.492938 | 174.1540141 |
| Asty66-028 | 7.646265897 | 5.235136659 | 10.05739514 | -4.822258477 | 333.5304059 | 123.6896625 | 543.3711493 | -419.6814868 | 309.762192 | 564.3344724 | 55.18991167 | 509.1445607 |
| Asty66-029 | 6.857569222 | 3.531821363 | 10.18331708 | -6.651495717 | 144.1535285 | 11.99185348 | 276.3152035 | -264.32335 | 527.1869099 | 789.9489786 | 264.4248411 | 525.5241375 |
| Asty66-030 | 10.40256402 | 10.47177166 | 10.33335637 | 0.138415293 | 198.6781934 | 79.95078523 | 317.4056016 | -237.4548164 | 414.4217143 | 488.090869 | 340.7525596 | 147.3383093 |
| Asty66-031 | 3.018212066 | 4.440555983 | 1.595868148 | 2.844687834 | 111.0144867 | 70.90632319 | 151.1226503 | -80.21632706 | 656.5840139 | 644.5243837 | 668.6436441 | -24.11926042 |
| Asty66-032 | 8.705738036 | 9.503788935 | 7.907687138 | 1.596101796 | 138.7185795 | 26.43963454 | 250.9975245 | -224.55789 | 567.8164283 | 719.3408711 | 416.2919854 | 303.0488857 |
| Asty66-033 | 4.044857943 | 1.941280144 | 6.148435742 | -4.207155598 | 150.987793 | 27.76665558 | 274.2089305 | -246.4422749 | 579.4739185 | 800.1953336 | 358.7525035 | 441.4428301 |
| Asty66-034 | 5.567201634 | 2.850138808 | 8.28426446 | -5.434125652 | 263.8388386 | 61.42462088 | 466.2530563 | -404.8284354 | 454.5493398 | 720.6963878 | 188.4022918 | 532.2940961 |
| Asty66-035 | 6.658118128 | 3.501640926 | 9.81459533 | -6.312954404 | 125.9895305 | 33.21029419 | 218.7687668 | -185.5584726 | 483.5582115 | 693.9321273 | 273.1842956 | 420.7478317 |
| Asty66-036 | 8.982398944 | 6.412600613 | 11.55219728 | -5.139596664 | 56.76266519 | 9.166805771 | 104.3585246 | -95.19171883 | 698.9298329 | 817.0879225 | 580.7717432 | 236.3161793 |
| Asty66-037 | 7.249903749 | 2.759729589 | 11.74007791 | -8.980348319 | 209.8383102 | 25.58391765 | 394.0927028 | -368.5087851 | 524.0302798 | 794.7106809 | 253.3498786 | 541.3608023 |
| Asty66-038 | 6.170313922 | 3.98214266 | 8.358485184 | -4.376342523 | 32.52036728 | 12.75303088 | 52.28770369 | -39.53467281 | 662.1447832 | 762.7738845 | 561.5156819 | 201.2582026 |
| Asty66-039 | 6.09161426 | 6.542529558 | 5.640698963 | 0.901830595 | 152.5904372 | 117.4431373 | 187.737737 | -70.29459975 | 513.8920168 | 564.553442 | 463.2305916 | 101.3228504 |
| Asty66-040 | 2.213233206 | 1.486573819 | 2.939892593 | -1.453318774 | 90.8745552 | 8.522411292 | 173.2266991 | -164.7042878 | 704.0071323 | 859.6714771 | 548.3427875 | 311.3286896 |
| Asty66-041 | 5.645599239 | 7.675361097 | 3.615837381 | 4.059523715 | 85.71453373 | 52.15493244 | 119.274135 | -67.11920258 | 625.4650486 | 642.2401575 | 608.6899398 | 33.55021773 |
| Asty66-042 | 11.79353291 | 12.2303039 | 11.35676191 | 0.873541991 | 244.6481227 | 171.748835 | 317.5474104 | -145.7985754 | 406.2381828 | 468.5087876 | 343.9675781 | 124.5412095 |
| Asty66-043 | 5.244420786 | 7.780061062 | 2.708780509 | 5.071280553 | 193.0604223 | 241.9183076 | 144.2025371 | 97.71577052 | 511.7089312 | 390.4286234 | 632.989239 | -242.5606156 |
| Asty66-044 | 8.961871138 | 3.497173778 | 14.4265685 | -10.92939472 | 95.84271755 | 11.57755008 | 180.107885 | -168.5303349 | 655.6518334 | 830.3803805 | 480.9232863 | 349.4570942 |
| Asty66-045 | 7.828399164 | 7.039001749 | 8.617796579 | -1.578794831 | 137.6793462 | 67.77263421 | 207.5860581 | -139.8134239 | 567.8897653 | 672.3994824 | 463.3800483 | 209.0194341 |
| Asty66-046 | 3.948355068 | 3.386010223 | 4.510699913 | -1.12468969 | 144.2995073 | 81.41822319 | 207.1807913 | -125.7625681 | 567.6687105 | 685.2588708 | 450.0785502 | 235.1803206 |
| Asty66-047 | 6.247853245 | 2.25528437 | 10.24042212 | -7.98513775 | 77.22201383 | 1.361083292 | 153.0829444 | -151.7218611 | 627.1924707 | 861.1437823 | 393.2411591 | 467.9026231 |
| Asty66-048 | 4.730619665 | 2.42217815 | 7.03906118 | -4.616883029 | 94.50526916 | 32.716745 | 156.2937933 | -123.5770483 | 560.9081299 | 700.9808411 | 420.8354187 | 280.1454224 |
| Asty66-049 | 6.816768935 | 4.749290666 | 8.884247205 | -4.134956539 | 108.8227116 | 44.20114531 | 173.4442779 | -129.2431326 | 561.8959238 | 665.8241579 | 457.9676896 | 207.8564683 |
| Asty66-050 | 4.528673682 | 5.209467968 | 3.847879396 | 1.361588571 | 115.8241574 | 44.2463299 | 187.4019849 | -143.155655 | 577.7621366 | 606.480785 | 549.0434883 | 57.43729671 |
| Asty66-051 | 5.840381534 | 1.914535539 | 9.766227528 | -7.851691988 | 55.66399753 | 5.742548188 | 105.5854469 | -99.84289869 | 725.1345096 | 864.5457954 | 585.7232237 | 278.8225718 |
| Asty66-052 | 4.475809657 | 2.541338035 | 6.410281279 | -3.868943244 | 19.96475633 | 15.45712371 | 24.47238896 | -9.01526525 | 704.7064422 | 699.7984081 | 709.6144762 | -9.816068104 |
| Asty66-053 | 8.499944853 | 3.789887932 | 13.21000177 | -9.420113843 | 99.66077109 | 3.876793208 | 195.444749 | -191.5679558 | 639.2607886 | 842.6468135 | 435.8747636 | 406.7720499 |
| Asty66-054 | 7.889708853 | 5.443397326 | 10.33602038 | -4.892623053 | 88.65288924 | 50.46018271 | 126.8455958 | -76.38541306 | 614.6153102 | 683.5919263 | 545.6386941 | 137.9532322 |
| Asty66-055 | 5.769715933 | 1.948002439 | 9.591429428 | -7.643426988 | 159.5790236 | 12.60844217 | 306.549605 | -293.9411629 | 566.8557446 | 838.6435049 | 295.0679842 | 543.5755207 |
| Asty66-056 | 2.495158766 | 2.708319963 | 2.281997569 | 0.426322393 | 219.5591424 | 130.7029249 | 308.4153598 | -177.7124349 | 527.2988268 | 654.984151 | 399.6135026 | 255.3706484 |
| Asty66-057 | 5.524989501 | 8.049740465 | 3.000238538 | 5.049501926 | 421.1065225 | 250.8856076 | 591.3274374 | -340.4418298 | 256.8266194 | 363.3028886 | 150.3503501 | 212.9525385 |
| Asty66-058 | 2.850121156 | 2.490195144 | 3.210047167 | -0.719852024 | 101.5352157 | 27.17995688 | 175.8904746 | -148.7105177 | 649.8877351 | 765.949283 | 533.8261872 | 232.1230958 |
| Asty66-059 | 12.07740035 | 10.76059837 | 13.39420233 | -2.633603958 | 90.65940933 | 58.88249356 | 122.4363251 | -63.55383154 | 634.7635841 | 681.2048161 | 588.3223521 | 92.88246406 |
| Asty66-060 | 10.17142658 | 10.63470062 | 9.708152543 | 0.926548077 | 172.274358 | 222.8137873 | 121.7349287 | 101.0788586 | 512.9859033 | 436.5977099 | 589.3740966 | -152.7763867 |
| Asty66-061 | 7.81833284 | 8.490731642 | 7.145934037 | 1.344797605 | 250.9676341 | 66.29615671 | 435.6391115 | -369.3429548 | 328.9821066 | 423.5527187 | 234.4114944 | 189.1412242 |
| Asty66-062 | 4.800140727 | 3.986602753 | 5.613678702 | -1.62707595 | 178.6380129 | 38.15065048 | 319.1253753 | -280.9747248 | 430.4710961 | 684.0674019 | 176.8747904 | 507.1926114 |
| Asty66-063 | 7.518992497 | 8.608513847 | 6.429471147 | 2.179042699 | 201.8782663 | 176.7788613 | 226.9776713 | -50.19881 | 436.5139453 | 412.9601835 | 460.0677072 | -47.10752371 |
| Asty66-064 | 4.474171612 | 2.333163944 | 6.61517928 | -4.282015336 | 163.3310388 | 31.33758735 | 295.3244902 | -263.9869028 | 524.7202067 | 790.033089 | 259.4073244 | 530.6257646 |
| Asty66-065 | 1.86154203 | 1.976081492 | 1.747002567 | 0.229078925 | 154.560463 | 23.29412719 | 285.8267989 | -262.5326717 | 501.9046828 | 796.0467412 | 207.7626244 | 588.2841168 |
| Asty66-066 | 4.078976497 | 1.560858653 | 6.597094341 | -5.036235689 | 121.3046374 | 3.043321042 | 239.5659538 | -236.5226327 | 589.2750389 | 838.8993162 | 339.6507617 | 499.2485544 |
| Asty66-067 | 12.16868903 | 13.18226441 | 11.15511365 | 2.027150759 | 101.6064673 | 64.64798029 | 138.5649544 | -73.9169741 | 544.7986183 | 624.8588884 | 464.7383482 | 160.1205402 |
| Asty66-068 | 9.334704725 | 8.71862084 | 9.950788611 | -1.232167771 | 177.4795632 | 126.5077583 | 228.4513682 | -101.9436099 | 499.7925009 | 554.8110594 | 444.7739424 | 110.037117 |
| Asty66-069 | 4.475373632 | 5.367543956 | 3.583203308 | 1.784340647 | 295.439884 | 154.2764991 | 436.6032689 | -282.3267698 | 317.3784899 | 481.3028302 | 153.4541496 | 327.8486806 |
| Asty66-070 | 5.467497322 | 6.784474082 | 4.150520563 | 2.633953519 | 69.44444417 | 13.30080044 | 125.5880879 | -112.2872875 | 623.6632462 | 727.666555 | 519.6599373 | 208.0066177 |
| Asty66-071 | 7.558624322 | 7.038037858 | 8.079210787 | -1.041172929 | 272.670935 | 103.8802697 | 441.4616003 | -337.5813306 | 361.3783921 | 537.0912576 | 185.6655267 | 351.4257309 |
| Asty66-072 | 6.725936536 | 8.741431195 | 4.710441878 | 4.030989316 | 326.6537364 | 272.6545979 | 380.6528749 | -107.998277 | 208.2513063 | 263.6115269 | 152.8910857 | 110.7204413 |
| Asty66-073 | 9.069422208 | 7.046806413 | 11.092038 | -4.045231591 | 165.5085641 | 88.17845677 | 242.8386714 | -154.6602146 | 466.7007296 | 616.1661662 | 317.2352931 | 298.9308731 |
| Asty66-074 | 5.147146325 | 4.4450244 | 5.849268249 | -1.404243849 | 120.8364611 | 51.46396379 | 190.2089584 | -138.7449946 | 530.6368881 | 671.6431731 | 389.630603 | 282.0125701 |
| Asty66-075 | 3.409864607 | 3.598135889 | 3.221593325 | 0.376542564 | 129.2865781 | 34.62142685 | 223.9517293 | -189.3303024 | 609.0493276 | 730.1704485 | 487.9282066 | 242.2422419 |
| Asty66-076 | 4.446073395 | 1.763854977 | 7.128291813 | -5.364436836 | 93.09309313 | 21.8968971 | 164.2892891 | -142.392392 | 641.3747087 | 818.9696643 | 463.7797531 | 355.1899112 |
| Asty66-077 | 6.653421803 | 4.016974313 | 9.289869294 | -5.272894982 | 122.617061 | 25.07090371 | 220.1632183 | -195.0923145 | 593.5459773 | 770.2049269 | 416.8870277 | 353.3178992 |
| Asty66-078 | 6.317334396 | 8.564791153 | 4.069877638 | 4.494913515 | 126.3583022 | 112.8858019 | 139.8308025 | -26.94500054 | 546.7109467 | 459.4525085 | 633.9693849 | -174.5168765 |
| Asty66-079 | 3.131309939 | 3.076996314 | 3.185623564 | -0.108627251 | 65.8582196 | 47.3765431 | 84.3398961 | -36.963353 | 637.4530777 | 638.2146017 | 636.6915537 | 1.523048 |
| Asty66-080 | 3.751664078 | 3.624237678 | 3.879090479 | -0.254852801 | 214.7814484 | 220.6421697 | 208.9207271 | 11.72144258 | 498.9413015 | 460.4312644 | 537.4513385 | -77.0200741 |
| Asty66-081 | 3.661289118 | 0.717559046 | 6.60501919 | -5.887460145 | 131.796727 | 3.447197104 | 260.1462568 | -256.6990597 | 622.2413385 | 844.202536 | 400.280141 | 443.922395 |
| Asty66-082 | 7.381144567 | 3.471571374 | 11.29071776 | -7.819146386 | 113.4822317 | 8.775442021 | 218.1890214 | -209.4135794 | 634.4448613 | 824.7546153 | 444.1351073 | 380.6195081 |
| Asty66-083 | 5.942618871 | 3.204806814 | 8.680430929 | -5.475624115 | 56.60382634 | 8.249221521 | 104.9584312 | -96.70920965 | 683.3187352 | 778.3700361 | 588.2674343 | 190.1026018 |
| Asty66-084 | 5.456691342 | 5.184566426 | 5.728816259 | -0.544249832 | 62.01896358 | 7.891918292 | 116.1460089 | -108.2540906 | 685.8042077 | 791.3357814 | 580.2726339 | 211.0631475 |
| Asty66-085 | 3.19750832 | 3.452868367 | 2.942148274 | 0.510720093 | 76.1740909 | 63.58024735 | 88.76793444 | -25.18768708 | 663.5006532 | 688.9500606 | 638.0512458 | 50.89881485 |
| Asty66-086 | 9.140756872 | 8.542329937 | 9.739183807 | -1.19685387 | 300.075423 | 285.5814163 | 314.5694298 | -28.98801342 | 349.4640464 | 361.8542138 | 337.0738791 | 24.78033473 |
| Asty66-087 | 7.387903215 | 4.246506967 | 10.52929946 | -6.282792497 | 172.9934795 | 8.634328438 | 337.3526305 | -328.718302 | 534.9884608 | 836.7179678 | 233.2589538 | 603.459014 |
| Asty66-088 | 5.206222871 | 1.656918516 | 8.755527227 | -7.098608711 | 113.2312867 | 2.079162458 | 224.383411 | -222.3042485 | 638.2917643 | 875.0006952 | 401.5828334 | 473.4178618 |
| Asty66-089 | 6.542887529 | 4.746721127 | 8.33905393 | -3.592332803 | 136.7051075 | 49.53773125 | 223.8724837 | -174.3347525 | 569.1507482 | 735.7211391 | 402.5803574 | 333.1407817 |
| Asty66-090 | 7.792867561 | 6.748340982 | 8.83739414 | -2.089053157 | 315.8759453 | 133.0017515 | 498.7501392 | -365.7483878 | 211.6494968 | 296.2413798 | 127.0576137 | 169.1837661 |
| Asty66-091 | 11.37598852 | 11.68214056 | 11.06983649 | 0.612304075 | 416.939509 | 490.6170064 | 343.2620116 | 147.3549948 | 247.6716294 | 159.6575739 | 335.6856849 | -176.0281109 |
| Asty66-092 | 3.65642903 | 5.364394041 | 1.948464019 | 3.415930021 | 294.9477264 | 390.6281304 | 199.2673225 | 191.3608079 | 376.0639101 | 260.1803185 | 491.9475018 | -231.7671833 |
| Asty66-093 | 10.29802425 | 10.2026244 | 10.3934241 | -0.190799707 | 190.4108969 | 140.8477927 | 239.974001 | -99.12620829 | 413.2465804 | 454.1444233 | 372.3487374 | 81.79568588 |
| Asty66-094 | 4.445790329 | 2.142420485 | 6.749160173 | -4.606739688 | 125.2613726 | 3.872622625 | 246.6501226 | -242.7774999 | 670.9125798 | 866.4824545 | 475.342705 | 391.1397495 |
| Asty66-095 | 6.816636959 | 7.557751131 | 6.075522787 | 1.482228345 | 287.9956344 | 257.123096 | 318.8681728 | -61.74507679 | 332.6958897 | 336.7610644 | 328.6307149 | 8.130349521 |
| Asty66-096 | 4.991130687 | 4.788561108 | 5.193700266 | -0.405139158 | 126.589089 | 16.60410423 | 236.5740738 | -219.9699695 | 420.4527449 | 706.8450396 | 134.0604503 | 572.7845893 |
| Asty66-097 | 5.017193395 | 3.439584439 | 6.594802351 | -3.155217912 | 55.3994963 | 18.7096819 | 92.08931071 | -73.37962881 | 643.5751728 | 764.7494723 | 522.4008734 | 242.348599 |
| Asty66-098 | 6.572411972 | 4.481956781 | 8.662867162 | -4.180910381 | 40.90757414 | 60.3839946 | 21.43115367 | 38.95284094 | 699.2760129 | 664.4102438 | 734.1417821 | -69.73153835 |
| Asty66-099 | 4.218787278 | 1.494916656 | 6.942657901 | -5.447741245 | 88.75611725 | 6.311172563 | 171.2010619 | -164.8898894 | 626.6241939 | 839.3101431 | 413.9382448 | 425.3718983 |
| Asty66-100 | 8.372544264 | 3.871936296 | 12.87315223 | -9.001215937 | 92.2060947 | 6.687937667 | 177.7242517 | -171.0363141 | 638.7456895 | 843.5421544 | 433.9492246 | 409.5929298 |
| Asty66-101 | 3.612785748 | 3.684155802 | 3.541415694 | 0.142740109 | 174.4862914 | 34.770187 | 314.2023959 | -279.4322089 | 472.463436 | 653.247692 | 291.67918 | 361.568512 |
| Asty66-102 | 8.587950168 | 10.07445959 | 7.101440747 | 2.973018843 | 181.6948889 | 139.4623792 | 223.9273987 | -84.4650195 | 476.1118756 | 457.6277651 | 494.595986 | -36.96822094 |
| Asty66-103 | 6.624373687 | 5.807835241 | 7.440912133 | -1.633076892 | 145.7262818 | 80.22188898 | 211.2306746 | -131.0087856 | 461.1656804 | 517.7594255 | 404.5719353 | 113.1874902 |
| Asty66-104 | 9.59450675 | 9.844057198 | 9.344956302 | 0.499100896 | 176.8024973 | 9.293320875 | 344.3116738 | -335.0183529 | 508.9805081 | 764.8328868 | 253.1281294 | 511.7047575 |
| Asty66-105 | 7.614379805 | 7.946057234 | 7.282702376 | 0.663354858 | 86.53792672 | 6.374429708 | 166.7014237 | -160.326994 | 632.3851638 | 759.5720724 | 505.1982552 | 254.3738173 |
| Asty66-106 | 8.729527803 | 9.353758062 | 8.105297544 | 1.248460518 | 75.3861495 | 48.08975625 | 102.6825428 | -54.5927865 | 683.2186356 | 681.6691686 | 684.7681026 | -3.098933979 |
| Asty66-107 | 11.37380651 | 10.97070034 | 11.77691269 | -0.806212346 | 175.8449415 | 104.1207871 | 247.5690959 | -143.4483089 | 507.7998143 | 597.8937294 | 417.7058992 | 180.1878302 |
| Asty66-108 | 6.694417561 | 6.256856531 | 7.13197859 | -0.875122059 | 423.0998351 | 384.1383039 | 462.0613664 | -77.92306256 | 217.7038154 | 288.5990158 | 146.808615 | 141.7904008 |
| Asty66-109 | 5.953768458 | 6.857362161 | 5.050174755 | 1.807187406 | 167.5446284 | 77.35721817 | 257.7320386 | -180.3748204 | 408.6430184 | 459.3127848 | 357.9732519 | 101.3395329 |
| Asty66-110 | 10.87757356 | 10.78128284 | 10.97386427 | -0.192581424 | 475.4799934 | 297.5725725 | 653.3874143 | -355.8148418 | 118.1914556 | 185.3353358 | 51.04757546 | 134.2877603 |
| Asty66-111 | 7.721540654 | 6.313595364 | 9.129485945 | -2.815890581 | 73.2204425 | 21.8767375 | 124.5641475 | -102.68741 | 714.1273219 | 800.6833224 | 627.5713213 | 173.1120011 |
| Asty66-112 | 4.313833624 | 4.580217572 | 4.047449676 | 0.532767896 | 260.9908535 | 166.395565 | 355.586142 | -189.190577 | 487.962615 | 549.342397 | 426.582833 | 122.759564 |
| Asty66-113 | 6.24515071 | 6.994305534 | 5.495995886 | 1.498309648 | 195.4840256 | 137.2789459 | 253.6891054 | -116.4101595 | 479.864935 | 516.121678 | 443.608192 | 72.51348602 |
| Asty66-114 | 10.28933087 | 10.50204603 | 10.07661571 | 0.425430325 | 177.268936 | 113.6761756 | 240.8616965 | -127.1855209 | 530.3584838 | 581.6086925 | 479.1082751 | 102.5004174 |
| Asty66-115 | 5.08240728 | 5.466502105 | 4.698312455 | 0.76818965 | 665.6399457 | 667.4876278 | 663.7922636 | 3.695364188 | 96.14579849 | 48.49015667 | 143.8014403 | -95.31128365 |
| Asty66-116 | 7.110073086 | 8.349323072 | 5.8708231 | 2.478499972 | 347.9316121 | 146.3345295 | 549.5286947 | -403.1941652 | 249.7462744 | 378.1232629 | 121.3692859 | 256.753977 |
| Asty66-117 | 6.363956605 | 5.801961437 | 6.925951772 | -1.123990335 | 192.7163263 | 89.3080575 | 296.1245951 | -206.8165376 | 462.1347042 | 553.2734124 | 370.9959959 | 182.2774165 |
| Asty66-118 | 7.795952796 | 8.57705109 | 7.014854501 | 1.562196589 | 285.663789 | 294.1045209 | 277.223057 | 16.88146388 | 394.4628661 | 352.7312059 | 436.1945262 | -83.46332027 |
| Asty66-119 | 9.568166706 | 9.755264208 | 9.381069203 | 0.374195004 | 94.19280426 | 87.40337554 | 100.982233 | -13.57885744 | 458.9141232 | 463.849266 | 453.9789804 | 9.870285583 |
| Asty66-120 | 6.075302642 | 6.591021759 | 5.559583525 | 1.031438234 | 128.2848827 | 110.6975048 | 145.8722606 | -35.17475585 | 672.7199416 | 691.1606036 | 654.2792795 | 36.88132412 |
| Asty66-121 | 9.769593578 | 10.67586178 | 8.863325375 | 1.812536405 | 436.8858438 | 329.9257595 | 543.8459281 | -213.9201686 | 164.6945558 | 188.4551203 | 140.9339914 | 47.52112896 |
| Asty66-122 | 7.403321499 | 7.712744676 | 7.093898322 | 0.618846354 | 159.9988879 | 107.9106893 | 212.0870866 | -104.1763973 | 437.3738323 | 527.9849302 | 346.7627343 | 181.2221959 |
| Asty66-123 | 9.199735571 | 13.09406379 | 5.305407351 | 7.788656441 | 100.2505974 | 91.42892794 | 109.0722668 | -17.64333883 | 574.6625088 | 508.0948979 | 641.2301197 | -133.1352218 |
| Asty66-124 | 5.09478274 | 2.683023729 | 7.506541752 | -4.823518023 | 141.6409468 | 2.569235917 | 280.7126576 | -278.1434217 | 578.397147 | 872.0157662 | 284.7785279 | 587.2372383 |
| Asty66-125 | 5.677126357 | 7.754904531 | 3.599348183 | 4.155556348 | 176.4511033 | 146.2080111 | 206.6941955 | -60.48618442 | 385.7482486 | 409.7409904 | 361.7555068 | 47.98548358 |
| Asty66-126 | 6.602361589 | 7.847573119 | 5.35715006 | 2.490423059 | 361.875416 | 135.2449659 | 588.5058661 | -453.2609002 | 282.6381933 | 415.8026078 | 149.4737789 | 266.3288289 |
| Asty66-127 | 10.14122223 | 10.56117127 | 9.721273195 | 0.839898077 | 329.0283342 | 291.7083748 | 366.3482935 | -74.63991873 | 319.6734921 | 330.401234 | 308.9457502 | 21.45548373 |
| Asty66-128 | 6.315798386 | 7.485634491 | 5.14596228 | 2.339672211 | 297.1631359 | 255.4443348 | 338.8819371 | -83.43760227 | 381.6865475 | 357.5047269 | 405.8683681 | -48.36364127 |
| Asty66-129 | - | - | - | - | - | - | - | - | - | - | - | - |
