# Supplementary material for: Genetic analysis reveals candidate genes for activity QTL in the blind Mexican tetra, Astyanax mexicanus
Source: PeerJ. 2018 Jul 18;6:e5189. doi: 10.7717/peerj.5189 (PMC6054784; doi:10.7717/peerj.5189)
Supplement: Table S2 — All members of the pedigree (n = 129) were scored for sex (1 = female, 0 = male), presence of albinism and presence of right and left eye (1 = present, 0 = absent) in a binary fashion. Eye and pupil size measurements (in pixels) were made from images where possible; in some cases an eye was technically present, but was too disorganized to measure the eye and/or pupil accurately. [file peerj-06-5189-s002.docx]

**Table S2: Sex, albinism and eye data for Asty66 F_2_ surface x Pachón hybrid pedigree.** All members of the pedigree (n = 129) were scored for sex (1 = female, 0 = male), presence of albinism and presence of right and left eye (1 = present, 0 = absent) in a binary fashion. Eye and pupil size measurements (in pixels) were made from images where possible; in some cases an eye was technically present, but was too disorganized to measure the eye and/or pupil accurately.

| Specimen | Sex | Albinism | Right Eye | Left Eye | Right Pupil | Left Pupil | Right Eye Area | Left Eye Area |
| --- | --- | --- | --- | --- | --- | --- | --- | --- |
| Asty66-001 | 1 | 0 | 1 | 1 | 10530 | 8583 | 61397 | 57146 |
| Asty66-002 | 1 | 0 | 1 | 1 | 10931 | 9646 | 52644 | 50421 |
| Asty66-003 | 1 | 0 | 1 | 1 | 12040 | 12085 | 53709 | 52138 |
| Asty66-004 | 1 | 0 | 1 | 1 | 16556 | 15157 | 80908 | 75006 |
| Asty66-005 | 0 | 0 | 1 | 1 | 12171 | 11042 | 45482 | 45360 |
| Asty66-006 | 0 | 0 | 1 | 1 | 10293 | 11055 | 39487 | 40970 |
| Asty66-007 | 1 | 0 | 1 | 1 | 14338 | 13416 | 59899 | 53822 |
| Asty66-008 | 0 | 0 | 1 | 1 | 12754 | 12571 | 59177 | 59308 |
| Asty66-009 | 1 | 0 | 1 | 1 | - | - | - | - |
| Asty66-010 | 1 | 0 | 1 | 1 | 13173 | 12059 | 64882 | 66087 |
| Asty66-011 | 1 | 0 | 1 | 1 | - | - | 42371 | 39638 |
| Asty66-012 | 0 | 0 | 1 | 1 | 6562 | 5582 | 33057 | 34579 |
| Asty66-013 | 1 | 0 | 1 | 1 | 14866 | 13815 | 73217 | 75362 |
| Asty66-014 | 0 | 0 | 1 | 1 | - | - | - | - |
| Asty66-015 | 0 | 0 | 1 | 1 | 13588 | 11268 | 60026 | 53979 |
| Asty66-016 | 1 | 0 | 1 | 1 | - | - | - | - |
| Asty66-017 | 0 | 0 | 1 | 1 | 11180 | 10743 | 55990 | 53825 |
| Asty66-018 | 1 | 0 | 1 | 1 | 7109 | 8050 | 30597 | 37801 |
| Asty66-019 | 1 | 0 | 1 | 1 | 7760 | 7313 | 44710 | 42482 |
| Asty66-020 | 1 | 0 | 1 | 1 | 16387 | 14830 | 81795 | 79853 |
| Asty66-021 | 0 | 0 | 1 | 1 | 13376 | 12565 | 52803 | 48975 |
| Asty66-022 | 1 | 0 | 1 | 1 | 15700 | 15529 | 77039 | 73945 |
| Asty66-023 | 1 | 0 | 1 | 1 | 9483 | 10644 | 53506 | 45213 |
| Asty66-024 | 1 | 0 | 1 | 1 | 10126 | 10148 | 58929 | 59620 |
| Asty66-025 | 1 | 0 | 1 | 1 | 12222 | 13558 | 60791 | 55919 |
| Asty66-026 | 0 | 0 | 1 | 1 | 5744 | 5962 | 34748 | 35940 |
| Asty66-027 | 1 | 0 | 1 | 1 | 16706 | 15507 | 60775 | 53335 |
| Asty66-028 | 0 | 0 | 1 | 1 | 8457 | 9604 | 40225 | 42390 |
| Asty66-029 | 1 | 0 | 1 | 1 | 4752 | 7275 | 37674 | 32054 |
| Asty66-030 | 0 | 0 | 1 | 1 | 8195 | 8190 | 33992 | 29952 |
| Asty66-031 | 1 | 0 | 1 | 1 | - | - | - | - |
| Asty66-032 | 1 | 0 | 1 | 1 | 8450 | 7117 | 50859 | 40261 |
| Asty66-033 | 1 | 0 | 1 | 1 | 17357 | 16646 | 72396 | 71512 |
| Asty66-034 | 0 | 0 | 1 | 1 | 11795 | 11903 | 57848 | 56702 |
| Asty66-035 | 0 | 0 | 1 | 1 | 4216 | 3508 | 35743 | 33154 |
| Asty66-036 | 0 | 0 | 1 | 1 | 11766 | 12884 | 60872 | 62075 |
| Asty66-037 | 1 | 0 | 1 | 1 | 5307 | 7217 | 28321 | 45083 |
| Asty66-038 | 0 | 0 | 1 | 1 | - | - | - | - |
| Asty66-039 | 0 | 0 | 1 | 1 | 15640 | 14691 | 64646 | 60485 |
| Asty66-040 | 1 | 0 | 1 | 1 | - | - | 41411 | 38768 |
| Asty66-041 | 0 | 0 | 1 | 1 | 6274 | 4291 | 29203 | 37840 |
| Asty66-042 | 0 | 0 | 1 | 1 | 13550 | 14317 | 67901 | 65818 |
| Asty66-043 | 0 | 0 | 1 | 1 | 7227 | 5708 | 42250 | 41810 |
| Asty66-044 | 0 | 0 | 1 | 1 | 8425 | 8899 | 44775 | 45584 |
| Asty66-045 | 0 | 0 | 1 | 1 | 12167 | 11694 | 55132 | 57682 |
| Asty66-046 | 0 | 0 | 1 | 1 | 20860 | 20243 | 86604 | 83838 |
| Asty66-047 | 0 | 0 | 1 | 1 | 11990 | 10948 | 51592 | 49364 |
| Asty66-048 | 1 | 0 | 1 | 1 | 11028 | 9966 | 46183 | 43592 |
| Asty66-049 | 1 | 0 | 1 | 1 | 11373 | 11456 | 61584 | 57901 |
| Asty66-050 | 0 | 0 | 1 | 1 | 4370 | 4366 | 31706 | 34125 |
| Asty66-051 | 0 | 0 | 1 | 1 | - | - | - | - |
| Asty66-052 | 0 | 0 | 1 | 1 | 14739 | 15012 | 56401 | 56512 |
| Asty66-053 | 0 | 0 | 1 | 1 | 13880 | 13580 | 50820 | 50032 |
| Asty66-054 | 0 | 0 | 1 | 1 | 5716 | 5566 | 35115 | 34977 |
| Asty66-055 | 1 | 0 | 1 | 1 | 9215 | 9756 | 56606 | 57175 |
| Asty66-056 | 0 | 0 | 1 | 1 | - | - | - | - |
| Asty66-057 | 1 | 0 | 0 | 0 | - | - | - | - |
| Asty66-058 | 1 | 0 | 0 | 0 | - | - | - | - |
| Asty66-059 | 1 | 0 | 0 | 0 | - | - | - | - |
| Asty66-060 | 1 | 0 | 1 | 1 | - | - | - | - |
| Asty66-061 | 0 | 0 | 0 | 0 | - | - | - | - |
| Asty66-062 | 0 | 0 | 1 | 1 | - | - | - | - |
| Asty66-063 | 1 | 0 | 1 | 1 | - | - | - | - |
| Asty66-064 | 0 | 0 | 1 | 1 | - | - | - | - |
| Asty66-065 | 1 | 0 | 0 | 1 | - | - | - | - |
| Asty66-066 | 0 | 0 | 1 | 1 | - | - | - | - |
| Asty66-067 | 0 | 0 | 1 | 1 | - | - | - | - |
| Asty66-068 | 0 | 0 | 0 | 0 | - | - | - | - |
| Asty66-069 | 1 | 0 | 0 | 1 | - | - | - | - |
| Asty66-070 | 1 | 0 | 1 | 1 | - | - | - | - |
| Asty66-071 | 1 | 0 | 1 | 1 | - | - | - | - |
| Asty66-072 | 0 | 0 | 0 | 0 | - | - | - | - |
| Asty66-073 | 0 | 0 | 1 | 0 | - | - | - | - |
| Asty66-074 | 1 | 0 | 1 | 1 | - | - | - | - |
| Asty66-075 | 1 | 0 | 0 | 0 | - | - | - | - |
| Asty66-076 | 1 | 0 | 1 | 1 | - | - | - | - |
| Asty66-077 | 1 | 0 | 1 | 1 | - | - | - | - |
| Asty66-078 | 0 | 0 | 1 | 1 | - | - | - | - |
| Asty66-079 | 1 | 0 | 1 | 1 | - | - | - | - |
| Asty66-080 | 0 | 0 | 1 | 1 | - | - | - | - |
| Asty66-081 | 1 | 0 | 1 | 1 | - | - | - | - |
| Asty66-082 | 0 | 0 | 1 | 1 | - | - | - | - |
| Asty66-083 | 0 | 0 | 1 | 1 | - | - | - | - |
| Asty66-084 | 0 | 0 | 1 | 1 | - | - | - | - |
| Asty66-085 | 1 | 0 | 1 | 1 | - | - | - | - |
| Asty66-086 | 0 | 0 | 1 | 0 | - | - | - | - |
| Asty66-087 | 0 | 1 | 1 | 1 | - | - | - | - |
| Asty66-088 | 0 | 0 | 1 | 1 | - | - | - | - |
| Asty66-089 | 0 | 0 | 1 | 1 | - | - | - | - |
| Asty66-090 | 0 | 0 | 1 | 1 | - | - | - | - |
| Asty66-091 | 0 | 0 | 1 | 1 | - | - | - | - |
| Asty66-092 | 0 | 0 | 1 | 1 | - | - | - | - |
| Asty66-093 | 0 | 1 | 0 | 0 | - | - | - | - |
| Asty66-094 | 0 | 0 | 1 | 1 | - | - | - | - |
| Asty66-095 | 1 | 0 | 1 | 1 | - | - | - | - |
| Asty66-096 | 1 | 1 | 1 | 1 | - | - | - | - |
| Asty66-097 | 0 | 0 | 1 | 1 | - | - | - | - |
| Asty66-098 | 0 | 0 | 1 | 1 | 16507 | 16132 | 74233 | 69511 |
| Asty66-099 | 0 | 0 | 1 | 1 | 8342 | 8001 | 37599 | 35817 |
| Asty66-100 | 0 | 0 | 1 | 1 | - | - | - | - |
| Asty66-101 | 1 | 1 | 0 | 0 | - | - | - | - |
| Asty66-102 | 1 | 1 | 1 | 1 | 5586 | 4566 | 31782 | 33017 |
| Asty66-103 | 1 | 1 | 1 | 1 | 8089 | 8132 | 40118 | 41105 |
| Asty66-104 | 0 | 1 | 1 | 1 | 9144 | 10499 | 54721 | 59577 |
| Asty66-105 | 0 | 1 | 1 | 1 | 5991 | 7017 | 36767 | 38054 |
| Asty66-106 | 0 | 1 | 0 | 0 | - | - | - | - |
| Asty66-107 | 0 | 1 | 1 | 1 | 5721 | 5202 | 45584 | 42682 |
| Asty66-108 | 1 | 1 | 1 | 1 | - | - | - | - |
| Asty66-109 | 1 | 1 | 1 | 1 | - | - | - | - |
| Asty66-110 | 0 | 1 | 1 | 1 | - | - | - | - |
| Asty66-111 | 0 | 1 | 1 | 1 | - | - | - | - |
| Asty66-112 | 0 | 1 | 1 | 1 | - | - | - | - |
| Asty66-113 | 0 | 1 | 1 | 1 | 9883 | 9811 | 47507 | 50993 |
| Asty66-114 | 0 | 1 | 1 | 1 | - | 730 | 18890 | 31111 |
| Asty66-115 | 1 | 1 | 1 | 1 | 13118 | 14501 | 54078 | 56009 |
| Asty66-116 | 1 | 1 | 0 | 1 | - | 2764 | - | 25166 |
| Asty66-117 | 1 | 0 | 0 | 0 | - | - | - | - |
| Asty66-118 | 1 | 0 | 0 | 0 | - | - | - | - |
| Asty66-119 | 0 | 0 | 0 | 1 | - | 1884 | - | 26899 |
| Asty66-120 | 0 | 0 | 0 | 0 | - | - | - | - |
| Asty66-121 | 1 | 0 | 1 | 1 | 5194 | 5485 | 41036 | 40745 |
| Asty66-122 | 0 | 0 | 0 | 0 | - | - | - | - |
| Asty66-123 | 0 | 0 | 0 | 0 | - | - | - | - |
| Asty66-124 | 1 | 0 | 0 | 0 | - | - | - | - |
| Asty66-125 | 1 | 1 | 1 | 1 | - | - | - | - |
| Asty66-126 | 0 | 1 | 1 | 1 | 5636 | 5655 | 43843 | 39129 |
| Asty66-127 | 0 | 0 | 1 | 0 | 7076 | - | 34090 | - |
| Asty66-128 | 1 | 0 | 0 | 0 | - | - | - | - |
| Asty66-129 | 1 | 0 | 1 | 1 | 5827 | 7613 | 50518 | 56949 |
