# Supplementary material for: Genetic analysis reveals candidate genes for activity QTL in the blind Mexican tetra, Astyanax mexicanus
Source: PeerJ. 2018 Jul 18;6:e5189. doi: 10.7717/peerj.5189 (PMC6054784; doi:10.7717/peerj.5189)
Supplement: Table S3 — Numbers indicate the number of genomic scaffolds anchored to both indicated linkage groups. [file peerj-06-5189-s003.docx]

**Table S3: Distribution of commonly-anchored genomic scaffolds between linkage maps published by Carlson *et al.* (2015) and O’Quin *et al.* (2013).** Numbers indicate the number of genomic scaffolds anchored to both indicated linkage groups.

|  |  | **Carlson *et al.* (2015) Linkage Groups** | | | | | | | | | | | | | | | | | | | | | | | | |
| --- | --- | --- | --- | --- | --- | --- | --- | --- | --- | --- | --- | --- | --- | --- | --- | --- | --- | --- | --- | --- | --- | --- | --- | --- | --- | --- |
|  |  | **1** | **2** | **3** | **4** | **5** | **6** | **7** | **8** | **9** | **10** | **11** | **12** | **13** | **14** | **15** | **16** | **17** | **18** | **19** | **20** | **21** | **22** | **23** | **24** | **25** |
| **O'Quin *et al.* (2013) Linkage Groups** | **1** | - | - | - | - | - | - | 1 | 1 | - | - | - | - | 18 | - | - | 1 | - | 1 | 1 | - | - | - | - | - | - |
|  | **2** | - | 1 | 16 | - | - | - | - | - | - | - | - | - | - | 1 | - | - | - | - | - | - | 1 | - | - | - | - |
|  | **3** | - | 1 | - | - | - | - | 10 | 5 | - | 1 | - | - | 2 | - | - | 1 | 1 | - | 1 | - | - | - | - | - | - |
|  | **4** | 1 | 1 | - | - | - | - | - | - | - | - | - | - | - | - | - | - | - | 1 | - | - | - | - | - | - | 16 |
|  | **5** | - | - | 1 | 1 | 1 | - | - | - | - | - | - | - | - | - | - | - | - | 5 | - | - | - | - | - | 1 | - |
|  | **6** | - | - | - | - | 1 | 1 | - | - | - | - | - | - | - | - | - | - | - | - | - | - | - | - | - | 10 | - |
|  | **7** | - | - | 1 | - | - | - | - | - | - | - | - | - | - | 11 | 1 | - | - | - | - | - | - | - | - | 1 | - |
|  | **8** | - | - | - | - | - | - | - | - | - | 16 | 8 | - | - | - | - | - | - | - | - | 1 | - | - | - | - | - |
|  | **9** | - | - | - | - | - | 1 | - | 1 | - | 1 | - | - | - | - | - | - | - | - | - | - | 3 | 1 | - | - | - |
|  | **10** | - | - | - | - | - | 1 | - | - | - | - | - | - | - | - | - | - | - | - | - | 9 | - | - | - | - | - |
|  | **11** | - | - | - | - | - | - | 1 | 1 | - | - | - | - | 1 | - | - | 1 | - | - | 12 | - | - | - | 1 | - | - |
|  | **12** | 6 | 5 | - | - | - | - | - | - | - | - | - | - | - | - | - | - | - | 1 | - | - | - | - | - | - | - |
|  | **13** | - | - | - | - | - | - | - | - | - | - | - | - | 1 | 1 | - | 11 | - | - | - | - | - | - | - | - | - |
|  | **14** | - | - | - | - | - | 1 | 1 | - | - | - | - | - | - | - | - | - | 1 | - | - | - | 1 | 3 | - | - | - |
|  | **15** | - | 1 | - | - | - | - | - | - | - | - | - | - | - | 1 | - | - | - | - | - | - | - | - | - | - | - |
|  | **16** | - | - | 1 | - | - | - | - | - | - | - | - | - | - | - | 1 | - | 5 | - | - | - | - | - | - | - | - |
|  | **17** | - | 1 | - | - | 1 | 14 | - | - | - | - | - | - | - | 1 | - | - | 1 | - | - | 1 | 1 | - | - | 1 | - |
|  | **18** | - | - | 1 | - | - | - | 1 | - | - | 1 | 1 | - | - | - | 9 | - | - | - | - | - | - | - | - | - | - |
|  | **19** | - | - | - | - | 1 | - | - | - | - | - | - | - | - | - | 1 | - | - | - | - | - | - | - | - | 1 | - |
|  | **20** | - | - | - | - | - | - | - | - | 5 | - | - | - | - | - | - | - | - | - | - | - | - | - | - | - | - |
|  | **21** | - | - | - | - | - | - | - | - | - | - | - | 6 | 2 | 1 | - | - | - | - | 1 | - | - | - | - | - | - |
|  | **22** | - | 1 | - | - | 10 | 1 | - | - | - | - | - | - | 1 | - | - | - | 1 | - | - | - | - | - | - | - | - |
|  | **23** | - | - | - | - | - | - | - | - | - | - | - | - | - | - | - | - | - | 1 | - | - | - | - | - | - | 1 |
|  | **24** | - | 1 | 1 | 5 | - | - | - | - | - | - | - | - | - | - | - | - | - | - | - | - | - | - | - | 1 | - |
|  | **25** | - | - | - | - | - | - | - | - | - | - | - | - | - | - | - | 1 | - | - | - | - | - | - | 5 | - | - |
